# Priming of Arabidopsis resistance to herbivory by insect egg deposition depends on the plant’s developmental stage

**DOI:** 10.1093/jxb/erac199

**Published:** 2022-05-06

**Authors:** Georgios Valsamakis, Norbert Bittner, Reinhard Kunze, Monika Hilker, Vivien Lortzing

**Affiliations:** Applied Zoology/ Animal Ecology, Institute of Biology, Freie Universität Berlin, Haderslebener Str. 9, 12163 Berlin, Germany; Applied Genetics, Institute of Biology, Freie Universität Berlin, Albrecht-Thaer-Weg 6, 14195 Berlin, Germany; Applied Genetics, Institute of Biology, Freie Universität Berlin, Albrecht-Thaer-Weg 6, 14195 Berlin, Germany; Applied Zoology/ Animal Ecology, Institute of Biology, Freie Universität Berlin, Haderslebener Str. 9, 12163 Berlin, Germany; Applied Zoology/ Animal Ecology, Institute of Biology, Freie Universität Berlin, Haderslebener Str. 9, 12163 Berlin, Germany; University of Birmingham, UK

**Keywords:** herbivory, insect eggs, larval feeding, plant ontogeny, plant resistance, plant tolerance, priming, salicylic acid, seed yield, transcriptome

## Abstract

While traits of plant resistance to herbivory often change during ontogeny, it is unknown whether the primability of this resistance depends on the plant’s developmental stage. Resistance in non-flowering *Arabidopsis thaliana* against *Pieris brassicae* larvae is known to be primable by prior egg deposition on leaves. We investigated whether this priming effect is maintained in plants at the flowering stage. Larval performance assays revealed that flowering plants’ resistance to herbivory was not primable by egg deposition. Accordingly, transcriptomes of flowering plants showed almost no response to eggs. In contrast, egg deposition on non-flowering plants enhanced the expression of genes induced by subsequent larval feeding. Strikingly, flowering plants showed constitutively high expression levels of these genes. Larvae performed generally worse on flowering than on non-flowering plants, indicating that flowering plants constitutively resist herbivory. Furthermore, we determined the seed weight in regrown plants that had been exposed to eggs and larvae during the non-flowering or flowering stage. Non-flowering plants benefitted from egg priming with a smaller loss in seed yield. The seed yield of flowering plants was unaffected by the treatments, indicating tolerance towards the larvae. Our results show that the primability of anti-herbivore defences in Arabidopsis depends on the plant’s developmental stage.

## Teaser

Arabidopsis resistance to herbivory can be primed by insect egg deposition during the vegetative stage of the plant, but this primability is lost in flowering plants at no reproductive cost.

## Introduction

A central paradigm in plant biology is that energy invested by a plant in its defences should trade-off with investments in growth and reproduction (Bazzaz *et al.*, 1987; Herms and Mattson, 1992; Huot *et al.*, 2014; Züst and Agrawal, 2017). Major defence strategies of plants against insect herbivores include resistance and tolerance mechanisms (Mauricio *et al.*, 1997; Núñez-Farfán *et al.*, 2007; Agrawal, 2011). Plants can resist attack through constitutive or inducible traits, which may directly impair the herbivore’s performance, or act indirectly by attracting natural enemies of a herbivore (Dicke and Hilker, 2003). Plants can also tolerate herbivory and compensate for tissue loss by herbivory through allocation of resources to different organs, thus maintaining their fitness in spite of being damaged (Strauss and Agrawal, 1999; Stowe *et al.*, 2000; Núñez-Farfán *et al.*, 2007; Fornoni, 2011).

Herbivore-induced plant resistance is mounted on demand and is thought to be less costly in the absence of herbivores than as a constitutive defence (Agrawal, 1998; Cipollini *et al.*, 2003; Steppuhn and Baldwin, 2008). However, the main drawback of inducible resistance is the time needed to establish effective defence measures against the herbivore (Steppuhn and Baldwin, 2008).

Priming (preparing) of inducible plant resistance using cues that reliably indicate herbivory can counterbalance this disadvantage (Martinez-Medina *et al.*, 2016; Hilker *et al.*, 2016). A plant’s response to priming cues may result in faster, earlier or stronger inducible resistance to the herbivore (Hilker *et al.*, 2016; Douma *et al.*, 2017). A wide range of cues has been shown to prime plant resistance against herbivores, including several volatile compounds, among them feeding-induced plant volatiles (Kost and Heil, 2006; Frost *et al.*, 2008; Arimura *et al.*, 2010; Dicke and Baldwin, 2010; Karban *et al.*, 2014), volatiles induced by insect egg depositions (Pashalidou *et al.*, 2020) and volatile insect sex pheromones that prime defence against feeding herbivores (Helms *et al.*, 2017) or against the herbivore’s eggs (Bittner *et al.*, 2019). In addition to these cues conveyed via air, there are others that are directly associated with the plant wound, i.e. damage-associated molecular patterns and oral larval secretions released into the plant wound that prime resistance against herbivory (e.g. Rasmann *et al.*, 2012; Heil and Land, 2014; Malook *et al.*, 2021). Furthermore, plants can be warned of impending larval feeding by insect egg depositions on their leaves (Hilker and Fatouros, 2015, 2016; Bandoly *et al.*, 2015; Geuss *et al.*, 2018).

Plant responses to priming cues can pay off and benefit reproduction. Several studies have shown that primed plants produce more seeds than non-primed plants (Karban and Maron, 2002; Karban *et al.*, 2012; Pashalidou *et al.*, 2015b). Furthermore, egg deposition can accelerate the formation of flowers (Lucas-Barbosa *et al.*, 2013). For non-primed plants, the effects of herbivory on the formation of plant reproductive organs range from herbivory-induced reduction in seed set, to the acceleration of flowering, to no effects on seed set through compensation of negative herbivory-induced effects (Strauss *et al.*, 2001; Schiestl *et al.*, 2014; Rusman *et al.*, 2020).

The ontogenetic stage of both the plant and the herbivore may shape the outcome of plant–herbivore interactions (Kant and Schuurink, 2021). For example, induced resistance traits are more prominent in juvenile plants, whereas constitutive resistance traits are more prominent in adult plants (Boege and Marquis, 2005; Barton and Koricheva, 2010). A meta-analysis by Quintero *et al.* (2013) revealed that increasing plant age was associated with decreasing indirect resistance mediated by herbivory-induced volatiles attracting parasitoids of the herbivore. In contrast, other types of indirect resistance—such as the provision of domatia for nesting ants—increased with advancing plant age. Changes in the type of defence strategies used in the course of a plant’s development are not limited to resistance against herbivory. The level of tolerance to herbivory may vary as well. Several studies report that plant tolerance to herbivory increases with plant age (Stowe *et al.*, 2000; Boege and Marquis, 2005). However, other studies have shown that tolerance does not change linearly with plant ontogeny, i.e. plants in intermediate stages were less tolerant than seedlings and mature plants (Del-Val and Crawley, 2005).

While plant resistance to herbivory is known to change throughout a plant’s development, it is unknown whether the primability of plant defence responses to herbivory is dependent on the plant’s developmental stage. Furthermore, we do not know whether plants in different developmental stages pay different reproductive costs for being primed, in terms of seed production.

We addressed these gaps in our knowledge by focusing on the priming of a plant’s defences against herbivores mediated by insect egg deposition. We used *Arabidopsis thaliana* (Brassicaceae) and the Large White Butterfly *Pieris brassicae* (Pieridae), as model species. Our previous studies have shown that Arabidopsis in its vegetative stage is more resistant to *P. brassicae* larvae when it has been primed by *P. brassicae* eggs prior to larval feeding. The improved resistance is evident in the reduced weight of consumed leaf tissue in previously egg-laden plants (Geiselhardt *et al.*, 2013; Lortzing *et al.*, 2019; Paniagua Voirol *et al.*, 2020; Valsamakis *et al.*, 2020). The primed, enhanced resistance of these plants during the vegetative stage relies on salicylic acid (SA)-mediated signalling and correlates with enhanced expression of certain marker genes, e.g. *PATHOGENESIS-RELATED GENE*s *PR5, PR2, PR1, PDF1.4* and *CATION EXCHANGER 3* (*CAX3),* in feeding-damaged, previously egg-laden plants, when compared with feeding-damaged, egg-free plants (Lortzing *et al.*, 2019; Valsamakis *et al.*, 2020).

Here, we treated Arabidopsis plants in their vegetative and reproductive stages, and of different chronological ages, with *P. brassicae* eggs. Egg-laden plants were then exposed to larval feeding. To assess for a priming effect of egg deposition on the plants’ resistance to hatching larvae, we measured the performance of larvae that fed on previously egg-laden or egg-free plants in either the vegetative or reproductive stage. We investigated whether egg-mediated priming of plant resistance against herbivores triggers alternative transcriptional reprogramming in plants in the vegetative and reproductive stages using RNA sequencing and qPCR. We then conducted experiments to disentangle whether differences in the effects of egg priming are dependent on the plant’s developmental stage or its chronological age. To understand whether plants in different developmental stages pay different costs for being primed, we quantified the total seed weight as a proxy for fitness in plants treated with eggs only, larval feeding only, or with both eggs and feeding. Priming exerted significant effects on seed production in plants treated in the vegetative stage. We addressed the question of whether this effect is due to different carbon/nitrogen allocation to its roots, or to different extent of feeding damage on egg-primed and non-primed plants.

Our results suggest that non-flowering plants take eggs as a warning to prepare their defences against impending larval herbivory. After transitioning to the flowering stage, the defences of plants against larval herbivory are no longer primable by prior egg deposition; instead, plants in the reproductive stage tolerate herbivory.

## Materials and methods

### Insect material

*Pieris brassicae* (L.) (Lepidoptera: Pieridae) adults were maintained in flight cages (45 × 45 × 60 cm) in a climate chamber under long day conditions (18 h light/6 h dark, 220 μmol m^-2^ s^-1^ light intensity, 23 °C, 70% relative humidity) and fed with 15% aqueous honey solution. To maintain rearing, female butterflies laid eggs on Brussels sprouts plants (*Brassica oleracea* var. *gemmifera*). Plants with eggs were maintained in a cage in a climate chamber (18 h light/6 h dark, 160 μmol m^-2^ s^-1^ light intensity, 20 °C and 70% relative humidity) until the larvae hatched 6 d after egg deposition. Larvae fed on Brussels sprouts throughout their development until pupation.

### Plant material

We used *Arabidopsis thaliana* (L.) Heynh. (Brassicales: Brassicaceae) ecotype Col-0 and the T-DNA-insertion line *svp-32* in the Col-0 background (obtained from Schubert group, Institute of Biology, Freie Universität Berlin). The *svp* mutant is deficient in expressing the short vegetative phase (*SVP*) transcription factor and flowers early (Liu *et al.*, 2009). Unless stated otherwise, plants grew on 3:1 mixture soil:vermiculite (Einheitserde Typ P soil; Kausek, Mittenwalde, Germany), as described in Firtzlaff *et al.* (2016). For further details of plant growth conditions, see ‘Experiments.’

### Plant treatments with Pieris brassicae eggs and larvae

We compared the egg-mediated priming responses to *P. brassicae* larvae in plants treated in the vegetative or the reproductive stage. Plants in the vegetative and those in the reproductive stage were treated with *P. brassicae* eggs (E), larval feeding (F), eggs and subsequent larval feeding (EF), or were left untreated as controls (C).

To treat plants with insect eggs, one butterfly deposited a clutch of approximately 40 eggs on one fully developed, non-senescent leaf per plant (in 5- to 7-week-old, non-flowering plants egg deposition took place on leaves in positions 14-17; in plants older than 7 weeks, butterflies laid eggs on leaves in positions 17-21). Each plant was treated with a different female, thus providing independent samples. To standardize the time that leaves had been exposed to the eggs, we carefully removed the eggs from leaves 6 d after deposition, just before the larvae were due to hatch. For egg removal, we used spring steel tweezers and a soft brush, and leaves were left undamaged. At this time, black head capsules of larvae were visible inside the eggs.

Feeding-damaged plants were prepared by exposing previously egg-laden or egg-free plants to 10 neonate larvae. The larvae hatched from eggs, which had been deposited on non-experimental Arabidopsis plants. One day before the larvae were due to hatch, the eggs were removed from those plants and transferred to Petri dishes until the larvae hatched. These larvae were then transferred onto the experimental plants. The transfer of larvae to previously egg-laden plants took place on the same day on which eggs had been removed from these plants.

The larvae were enclosed in a clip cage (2 cm in diameter, 1.7 cm high), which was fixed to the leaf on which the egg clutch had been laid. The leaves of untreated controls and of egg-treated plants without feeding damage were likewise covered with empty clip cages. After the larvae had fed for 2 d within a clip cage, the cages were removed, and larvae were allowed to feed on the entire plant until almost all leaf material had been consumed (only the midribs of leaves were left). To avoid larval escape, the plants were placed in PLEXIGLAS® cylinders (14.5 cm diameter, 15 cm high) with gauze lids.

In nature, larvae move from leaves to the flowers and prefer feeding upon flowers rather than upon leaves (Smallegange *et al.*, 2007). Therefore, we assessed whether egg-laden leaves were systemically primed for greater resistance against larvae that feed on flowers. For this experiment, only five, rather than 10 (as for leaves), neonate larvae were transferred to the flowers of a plant with an egg-laden or egg-free leaf, to ensure those larvae would have enough material to feed upon for 2 d. The flowers were covered with fine mesh bags (7 × 10 cm) for the duration of the feeding period.

For all experiments, replicates were arranged in randomized complete blocks, each block with size-matched plants. Hence, each group of replicates represented one block.

### Experiments

An overview of the experiments is provided in Table 1. We refer to plants in the vegetative stage as ‘non-flowering plants’ and to plants in the reproductive stage as ‘flowering plants.’ Unless stated otherwise, plants were kept in climate chambers under short day conditions (SD; 8 h light/16 h dark, 120 μmol m^−2^ s^−1^) or long day conditions (LD; 16 h light/8 h dark, 120 μmol m^−2^ s^−1^ light intensity); the temperature in the climate chambers was always 20 °C, and relative humidity was 50%. The experiments addressed the following questions:

**Table 1.** Overview of experiments conducted and their readouts. One week before plants were treated with eggs or no eggs, the plants were acclimatized for 1 week under short day conditions with 8 h/16 h light/dark cycle. Q: question, Exp.: experiment, NFL: non-flowering plants, FL: flowering plants.

**Q**	**Exp.**	**Light/dark cycle (hours)**	**Chronological plant age (weeks)**	**Plant stage (NFL/FL)**	**Readout**
**1**	1	8/16	5	NFL	Weight of 2-day-old larvae (LW 2 d)
	2	8/16	7	NFL	LW 2 d, 6 d
	3	8/16	9	FL	LW 2 d, 5 d
	4	8/16	12	FL	LW 2 d, 14 d
	5	8/16	12	FL	LW 2 d (on flowers)
	6^a^	8/16 16/8	10	FL	LW 2d, 12d
**2**	7	8/16	7	NFL	RNA-seq
		8/16	12	FL	
**3**	8	10/14	11	FL	LW, 2 d qPCR from untreated control plants
		6/18	11	NFL	
**4**	9	16/8	7	FL	LW 2 d, 6 d, qPCR after 2 d of feeding
		8/16	7	NFL	
		8/16	7*(svp-32)*	FL	
**5**	10^b^	8/16	7	NFL	Seed weight (SW)
		8/16	12	FL	
**6**	11^c^	8/16	7	NFL	C/N concentration
**7**	12	8/16	7	NFL	SW after recovery from feeding + cutting

Plants were exposed for 7 weeks to short day conditions with an 8 h/16 h light/dark cycle, after which the photoperiod length was steadily increased until long day conditions with a 16 h/8 h light/dark cycle were reached.

Experiments were conducted in climate chambers. The experiment on seed production in non-flowering plants was repeated under greenhouse conditions (for details see ‘Materials and Method’ section).

Arabidopsis plants grew under hydroponic culture conditions.

**Fig. 1.**
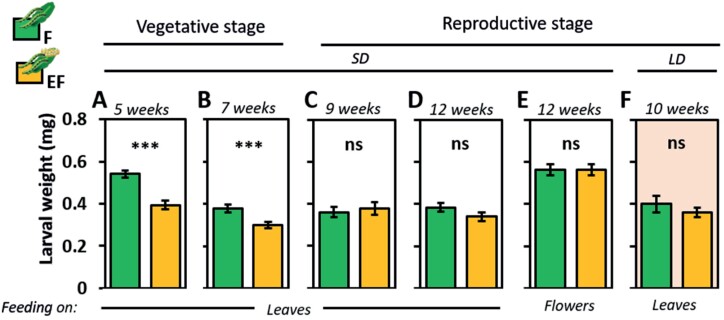
Impact of *Pieris brassicae* eggs on non-flowering and flowering Arabidopsis on *P. brassicae* larval weight. Larval weight in mg (means ±SE) of larvae after 2 d feeding on previously egg-laden (EF, yellow) or egg-free (F, green) plants in the vegetative (left side) or reproductive stage (right side). Chronological age of plants when treated with eggs: (A) 5 weeks old; (B) 7 weeks old; (C) 9 weeks old, (D, E): 12 weeks old (performance on leaves and flowers); (F) 10 weeks old. (A-E) Plant growth and treatment under short day conditions (8 h/16 h light/dark cycle). (F) Plants grew for 7 weeks under short day conditions and were treated under long day conditions (16 h/8 h light/dark cycle; see Table 1, Exp. 1-6). Statistically significant differences between treatments (***P<0.001) and non-significant differences (‘ns’, *P*>0.05) are shown, as assessed with Student’s *t*-test; *n*=7–10 (plants) per treatment. Statistical details and data for larval weight measurements at different time points are listed in Supplementary Table S3.

**Question 1:** Do flowering and non-flowering plants respond similarly to insect eggs in their primed resistance against larvae? The plants used for addressing this question were in different developmental stages (i.e. vegetative versus reproductive), but were also of different chronological ages (i.e. the number of weeks after being sown). We investigated the performance of *P. brassicae* larvae on plants in their vegetative phase, which grew in climate chambers for either 5 or 7 weeks under SD before they were treated with eggs (**Exp. 1, 2**), and on plants in their reproductive stage, which grew for either 9 or 12 weeks under the same SD conditions prior to treatment with eggs (**Exp. 3, 4**). To test whether egg deposition on leaves induced systemic priming effects in flowers, the butterflies laid eggs on the leaves of 12-week-old plants that had grown under SD conditions, as described above (**Exp. 5**). For each plant age, we conducted independent experiments using different sets of plants. Additionally, we investigated larval performance on 10-week-old flowering plants under LD conditions (**Exp. 6**). For this, 7-week-old plants (first grown under SD) were exposed to steadily increasing light periods (a 15 min longer period of light each day) until LD conditions were reached, and plants were 10-week-old. The data for larval performance are presented in Fig. 1.

**Fig. 2.**
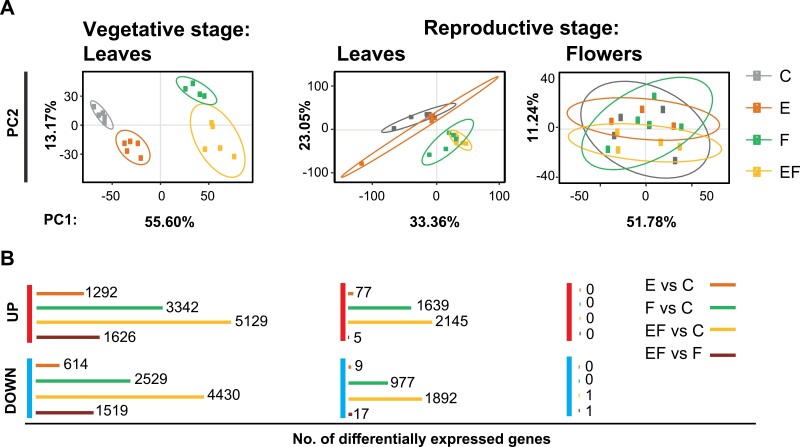
Impact of *Pieris brassicae* eggs on the transcriptomes of feeding-damaged or non-feeding-damaged leaves or flowers of Arabidopsis plants in the vegetative or reproductive stage. (A) Scatter plots of PCA showing group patterns of samples from each treatment according to the first two principal components. Ellipses indicate 95% confidence intervals. (B) Number of differentially expressed genes (red: UP: up-regulated, blue: DOWN: down-regulated) for the following treatment comparisons: E versus C (orange), F versus C (green), EF versus C (yellow) and EF versus F (dark red). The length of the lines depicts the differences in the number of differentially expressed genes; *n*=3–5 samples per treatment. Plants in the vegetative stage were 7 weeks old; those in the reproductive stage were 12 weeks old (see Table 1, Exp. 7). The plants were exposed to *P. brassicae* eggs (E, orange), feeding for 1 d by *P. brassicae* larvae (F, green), egg deposition followed by feeding for 1 d (EF, yellow), or were left as untreated controls (C, grey).

**Fig. 3:**
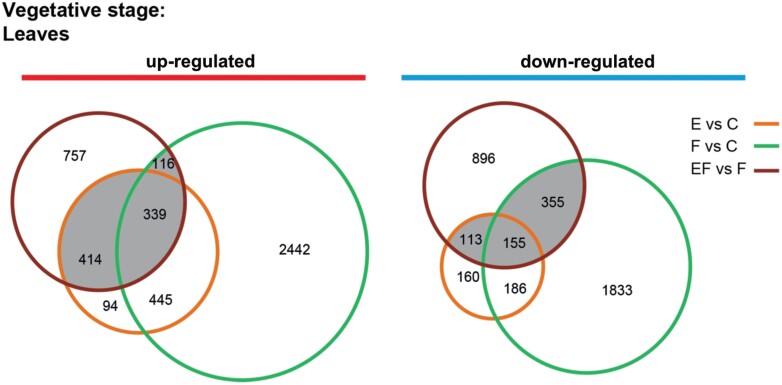
Transcriptional response of non-flowering Arabidopsis plants (vegetative stage) to *Pieris brassicae* eggs and larval feeding. Plants in the vegetative stage were 7 weeks old; those in the reproductive stage were 12 weeks old (see Table 1, Exp. 7). The comparisons include up-regulated (red) and down-regulated genes (blue) of the transcriptional response to the eggs (E versus C, orange), the response to 1 d of larval feeding (F versus C, green) and the response to larval feeding on leaves with and without prior egg deposition (EF versus F, dark red).

**Fig. 4.**
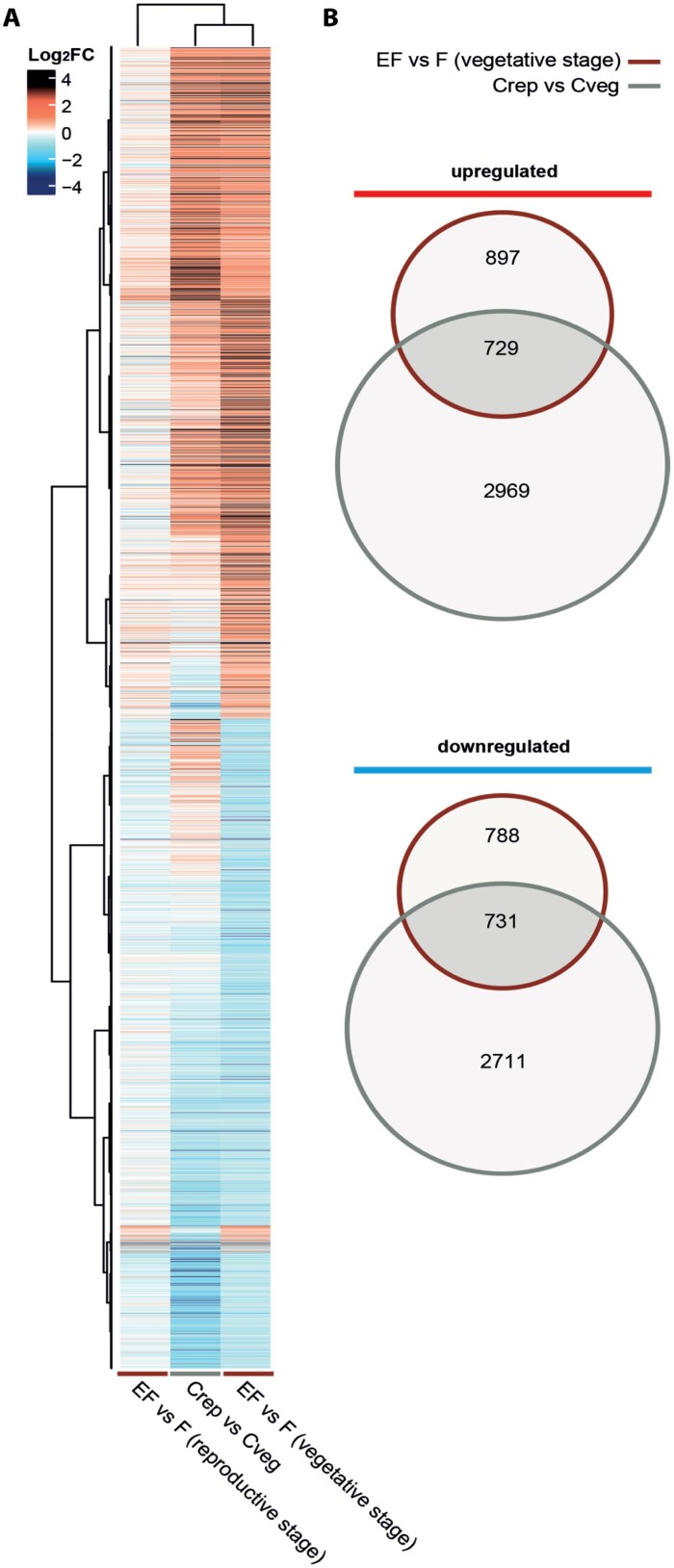
Characterization of the transcriptional priming response of Arabidopsis plants in the vegetative and reproductive stages. (A) Heatmaps of the transcriptional priming response (EF versus F) of non-flowering plants (vegetative stage) compared with (i) the transcriptional changes of the same genes in similarly treated flowering plants (EF versus F reproductive stage) and (ii) the transcriptional changes of the same genes when comparing untreated control leaves from flowering plants to untreated control leaves from non-flowering plants (Crep versus Cveg). (B) Venn diagrams showing the number of commonly up-regulated and down-regulated genes in the differentially expressed transcriptome of egg-laden, feeding-induced, non-flowering plants in the vegetative stage (EF versus F vegetative stage) and the differentially expressed transcriptome of untreated plants in the reproductive stage (Crep versus Cveg). Plants in the vegetative stage were 7 weeks old; those in the reproductive stage were 12 weeks old (see Table 1, Exp. 7). Plants were treated with *Pieris brassicae* larval feeding (F), eggs and subsequent larval feeding (EF), or were left as untreated controls (C). Log_2_FC in expression is shown in red = up-regulation, blue = down-regulation.

**Question 2**: How do the transcriptomes of non-flowering and flowering plants differ according to the treatments they are exposed to? For RNA-seq studies, 7- and 12-week-old non-flowering and flowering plants, respectively, were each either treated with eggs or left without eggs, followed by larval feeding damage, or left undamaged. The plants grew under SD, as described above (**Exp. 7**). To harvest leaf material and flowers from the 7- and 12-week-old plants simultaneously, the 7-week-old plants were sown 5 weeks later than the 12-week-old plants. RNA-seq data are presented in Figs 2–4.

**Questions 3 and 4**: Is the egg-mediated priming effect dependent on the plant’s chronological age (i.e. the number of weeks after being sown) or on the plant’s developmental stage? We conducted two different experiments, one with flowering and non-flowering plants of a ­chronological age of 11 weeks (Q3, **Exp. 8**), and another experiment with younger flowering and non-flowering plants of a chronological age of 7 weeks (Q4, **Exp. 9**).

**Fig. 5.**
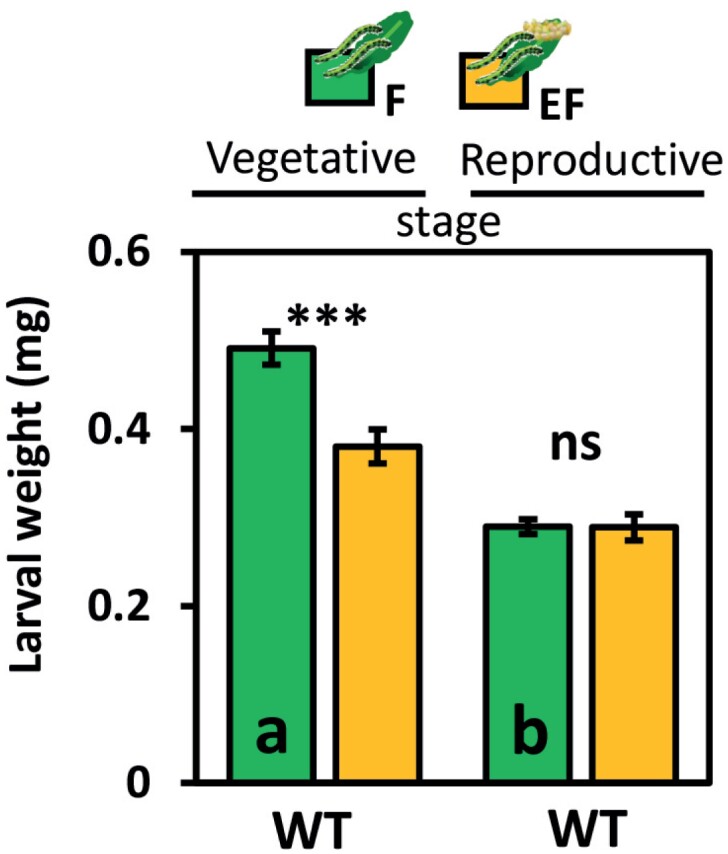
Impact of *Piers brassicae* eggs on 11-week-old flowering and non-flowering Arabidopsis plants (see Table 1, Exp. 8). Larval weight in mg (means ±SE) of *P. brassicae* larvae after 2 d feeding on previously egg-laden (EF, yellow) or egg-free (F, green), non-flowering (left side) or flowering (right side), wild-type (WT) plants. Significant differences between treatments (***P<0.001) and non-significant differences (‘ns’, *P*>0.05, Student’s *t*-test) are shown. Different lowercase letters indicate significant differences between larval weights on egg-free flowering and non-flowering WT (*P*<0.001, Student’s *t*-test); *n*=8 plants per treatment. Statistical details are provided in Supplementary Table S3.

**Fig. 6.**
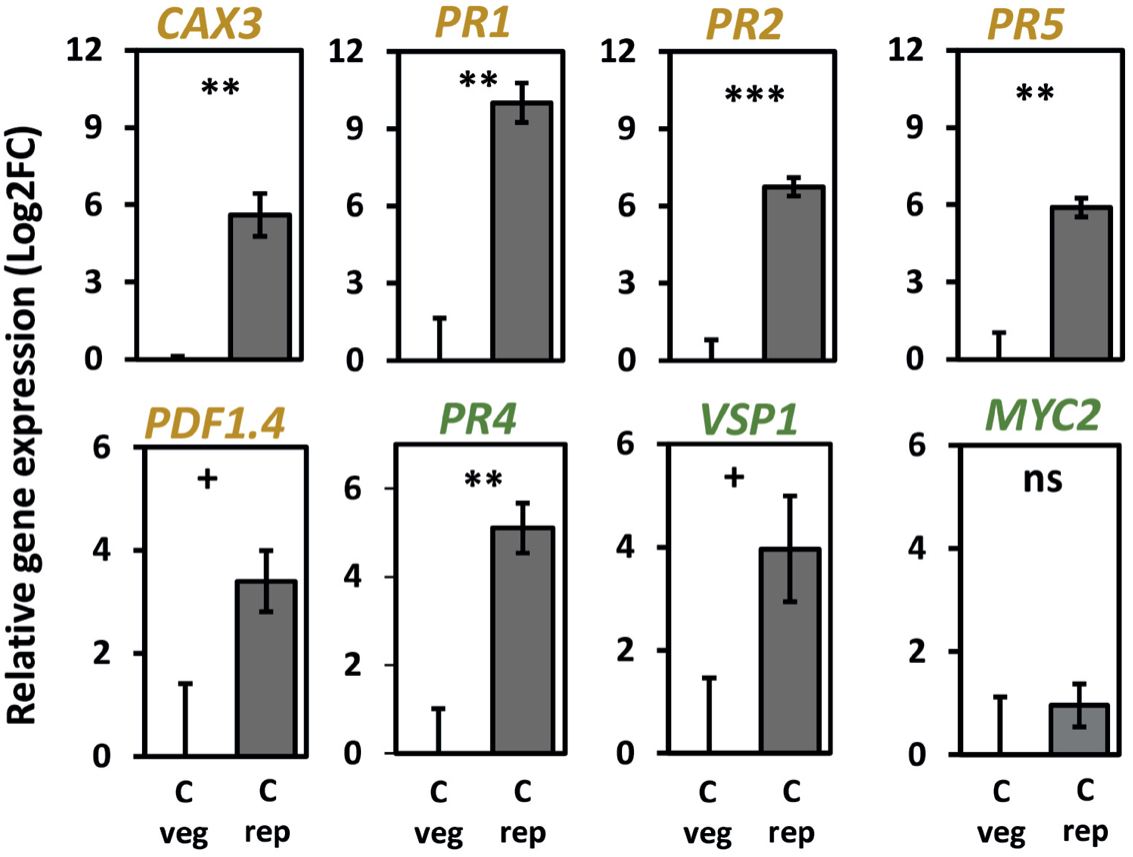
Transcriptional response of flowering and non-flowering Arabidopsis plants of identical chronological age, i.e. 11 weeks old (see Table 1, Exp. 8). Expression level (Log_2_FC, means ±SE) of priming marker genes (*PR5*, *PR2*, *PR1*, *CAX3*, *PDF1.4*) and jasmonic acid- or ethylene-responsive genes (*MYC2*, *VSP1*, and *PR4*) in untreated, non-flowering and flowering wild-type plants [Cveg and Crep, respectively] are shown. Significant differences are indicated between treatments (**P<0.01, ***P<0.001), differences by trend (+, *P*<0.1) and non-significant differences (‘ns’, *P*>0.1), as assessed with Student’s *t*-test; *n*=8–10 (plants) per treatment. Statistical details are provided in Supplementary Table S2. C: untreated control; veg: plants in the vegetative stage; rep: plants in the reproductive stage.

To obtain 11-week-old flowering plants for Exp. 8, the plants were grown under SD with a 10 h/14 h light/dark cycle. To obtain 11-week-old non-flowering plants, the 2-week-old plants were transferred to SD with a 6 h/18 h light/dark cycle for 8 weeks. Before plants were treated with eggs, they acclimatised for 1 week under SD with an 8 h/16 h light/dark cycle. Larval performance was tested on egg-laden or egg-free flowering and non-flowering 11-week-old plants. The data are presented in Fig. 5. In addition, we took samples from untreated 11-week-old flowering and non-flowering plants for qPCR analyses. These data are presented in Fig. 6.

**Fig. 7.**
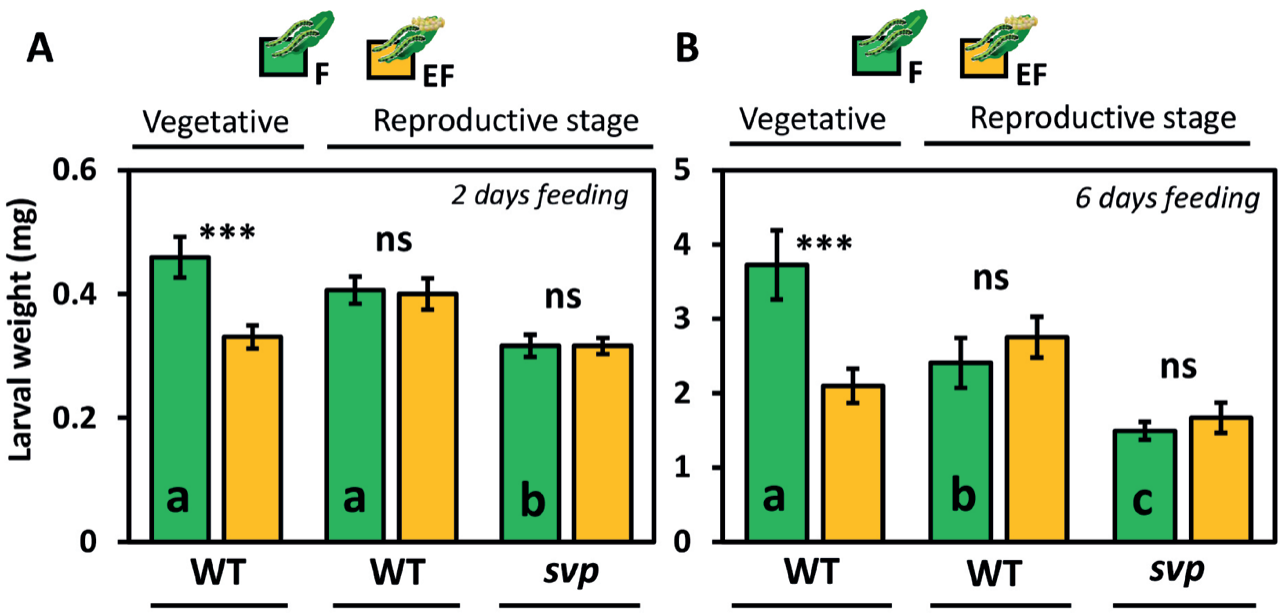
Impact of *Pieris brassicae* eggs on 7-week-old flowering and non-flowering Arabidopsis plants (see Table 1, Exp. 9). (A, B) Larval weight in mg (means ±SE) of *P. brassicae* larvae after (A) 2 d, and (B) 6 d of feeding on previously egg-laden (EF, yellow) or egg-free (F, green), non-flowering (left side) or flowering (right side), plants. Significant differences between treatments (***P<0.001) and non-significant differences (‘ns’, *P*>0.05, Student’s *t-*test) are shown. Different lowercase letters indicate significant differences between larval weights on untreated controls of non-flowering, flowering WT, and *svp-32,* plants (*P*<0.05, Student’s and Welch *t-*test). *P* values were *FDR* corrected; *n*=8 (plants) per treatment. Statistical details are provided in Supplementary Table S3.

**Fig. 8.**
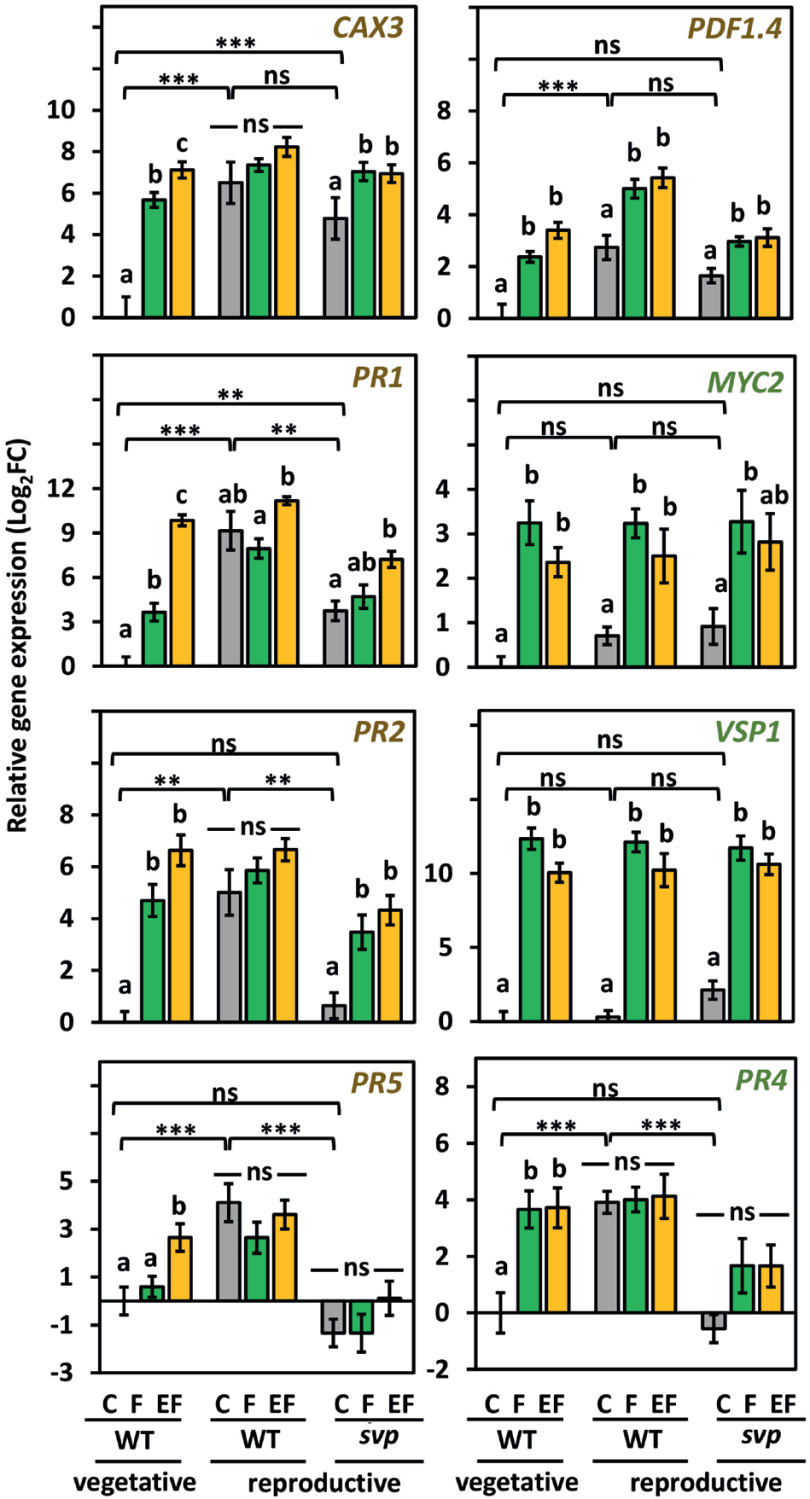
Transcriptional response of flowering and non-flowering Arabidopsis plants of identical chronological age, i.e. 7 weeks old (see Table 1, Exp. 9). Expression level (Log_2_FC, means ±SE) of priming marker genes (*PR5*, *PR2*, *PR1*, *CAX3*, *PDF1.4*) and jasmonic acid- or ethylene-responsive genes (*MYC2*, *VSP1* and *PR4*) in flowering and non-flowering wild-type (WT) and flowering *svp-32* mutant plants after treatment with *Pieris brassicae* larval feeding with (EF) or without (F) prior *P. brassicae* egg deposition, or plants were left untreated (C). Different letters above the bars indicate significant differences between the treatments for each WT stage and *svp-32* (*P*<0.05; ANOVA with Tukey’s test post-hoc); Significant differences are shown between untreated controls of non-flowering WT compared to flowering WT and *svp-32* (**P<0.01, ***P<0.001) and non-significant differences (‘ns’, *P*>0.05), as assessed with Student’s *t*-test and *FDR P* value adjustment; *n*=7–8 (WT plants) and 5–8 (*svp-32*) plants per treatment. Statistical details are provided in Supplementary Table S2.

To obtain 7-week-old flowering plants for Exp. 9, plants were germinated under SD with 8 h/16 h light/dark cycle and 2 weeks later they were transferred to LD conditions where they grew for 4 weeks. Before plants were treated with eggs, they acclimatised for 1 week under SD with a 8 h/16 h light/dark cycle. To obtain 7-week-old non-flowering plants, they were grown under SD with 8 h/16 h light/dark cycle. Larval performance and gene expression (via qPCR) were determined on egg-laden and egg-free flowering and non-flowering 7-week-old plants. The data are presented in Figs 7, 8. Additionally, we tested the performance of larvae on previously egg-laden and egg-free early flowering *svp-32* mutant plants, which were also grown for 7 weeks under SD, as described above. In contrast to wild-type (WT) plants, *svp* mutant plants flower at the chronological age of 5 weeks, even when kept under SD (Hartmann *et al.* 2000).

**Fig. 9.**
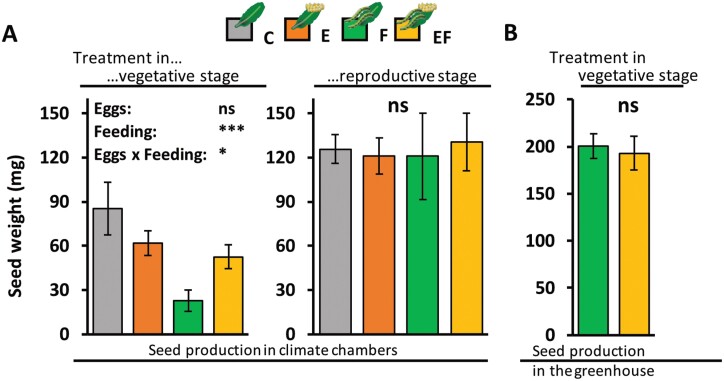
Impact of *Pieris brassicae* egg deposition and larval feeding damage on total seed weight of subsequently regrown Arabidopsis plants. (A) Treatments of leaves with eggs and feeding in the vegetative, non-flowering plant stage (left, 7-week-old plants) or in the reproductive, flowering plant stage (right, 12-week-old plants). Plants produced seeds in climate chambers under long day conditions (see Table 1, Exp. 10). The treatments effects were statistically evaluated with a linear mixed model with eggs, feeding and their interaction as fixed factors and experimental block as random factor. The asterisks represent significant effects of the factors (***P<0.001, *0.01<*P*<0.05), non-significant differences (ns, *P*>0.05); *n*=6–10 (plants) per treatment. (B) Plants were treated in the vegetative stage and were 7 weeks old. In contrast to the experiment shown in Fig. 9A, the leaf tissue that had been left after larval feeding was cut away. After recovery, plants produced seeds in the greenhouse under long day conditions (see Table 1, Exp. 12). Total seed weight per plant in mg (means ±SE); plants were treated with *P. brassicae* eggs (E, orange bars), *P. brassicae* larval feeding (F, green bars), with eggs followed by feeding (EF, yellow bars), or were left untreated as controls (C, grey bars). Non-significant differences (‘ns’ *P*>0.05) are shown, as assessed with Student’s *t*-test; *n*=9–10 (plants) per treatment. Statistical details are listed in Supplementary Table S2.

**Question 5**: How does the priming of anti-herbivore defences by egg deposition affect seed production in non-flowering and flowering Arabidopsis? We used 7- and 12-week-old plants grown under SD in a climate chamber (**Exp. 10**). To treat 7- and 12-week-old plants with eggs simultaneously, 12-week-old plants were sown 5 weeks earlier than the 7-week-old plants. After the treatments, the feeding-damaged plants were allowed to recover and regrow for 1 week. After this, damaged (F- and EF-plants) and undamaged (C- and E-plants) plants were exposed to LD for seed production (data presented in Fig. 9A). The seeds were collected when plants reached full maturity.

The experiment on the effect of egg deposition and feeding damage on seed production was repeated, but plants were transferred to the greenhouse for seed production after larval feeding; here, they were exposed to 16 h light/ 8 h dark, 100-150 μmol m^−2^ s^−1^ light intensity and 20–24 °C, instead of being kept in a climate chamber under LD conditions (Supplementary Fig. S1).

**Question 6:** Is the beneficial effect of prior egg deposition on seed weight in feeding-damaged, 7-week-old plants treated in the vegetative stage due to resource allocations to the roots? We determined the carbon (C) and nitrogen (N) contents in the roots (for details of the quantification method see ‘Determination of carbon (C) and nitrogen (N) contents in Arabidopsis*’*; **Exp. 11**). Plants were grown in a hydroponic culture system (details described in ‘Hydroponic culture of Arabidopsis’) under SD. Data are presented in Table 2.

**Table 2.** Allocation of carbon and nitrogen to the roots of Arabidopsis during its vegetative stage (7-week-old) in response to *Pieris brassicae* egg deposition and/or larval feeding (Exp. 11, Table 1).

**Treatment**	**C%**	**N%**	**C:N**	**No. of biological replicates**
C	40.38 ± 0.98	5.08 ± 0.10	8.20 ± 0.36	6
E	39.59 ± 0.50	5.37 ± 0.14	7.87 ± 0.36	7
F	38.87 ± 0.62	5.35 ± 0.21	7.89 ± 0.47	8
EF	39.80 ± 0.89	5.15 ± 0.15	8.70 ± 0.43	8

Data are shown as means ±SE of percentage of carbon (C%) and nitrogen (N%) and the C:N ratio of dry roots of plants subjected to four plant treatments: untreated controls (C), plants exposed to *P. brassicae* eggs (E), plants fed upon by *P. brassicae* larvae for 7 d (F), or plants exposed to eggs and subsequent feeding by larvae for 7 d (EF). Statistical differences were tested with ANOVA and post-hoc Tukey tests. There were no significant differences between the treatments for the variables tested (*P*>0.05). The number of replicates (plants) was n=6–8 per treatment.

**Question 7:** Is the beneficial effect of prior egg deposition on seed yield in non-flowering, feeding-damaged, 7-week-old plants due to there being less leaf damage inflicted by larvae on previously egg-laden plants, compared with egg-free plants of an equivalent age (**Exp. 12**)? To expose plants to the same extent of leaf damage, we cut the leaf material that remained after 6 d of larval feeding from both egg-laden and egg-free plants. The plants were grown under SD. After the plants had recovered from cutting, they were transferred to the greenhouse to flower and produce seeds. The seeds were collected when plants had reached full maturity, and were weighed (compared with Question 5). Data are presented in Fig. 9B.

### Sampling of plant material for transcript analyses and total RNA extraction

For RNA-seq, leaf material (and flowers) from feeding-damaged plants (F and EF) was harvested 1 d after feeding; for quantitative real-time PCR it was harvested 2 d after feeding. Samples from plants that had not been subjected to feeding damage (C and E) were harvested at the same time as feeding-damaged plants. In addition to leaf material for RNA-seq, we harvested flowers to assess whether this tissue was systemically primed by egg deposition on leaves. The harvested samples were immediately frozen in liquid nitrogen and stored at –80 °C.

Total RNA was extracted according to the protocol described by Oñate-Sánchez and Vicente-Carbajosa (2008). Following RNA extraction, samples were treated with the TURBO DNA free™ kit (ThermoFisher Scientific, Waltham, USA) to remove genomic DNA.

Samples for RNA-seq were mixed with 0.1 volume of 3 M NaOAc (pH 5.5), followed by the addition of 2 volumes of ethanol prior to shipping on dry ice for RNA sequencing at Macrogen Inc. (Seoul, South Korea).

### RNA quality assessment and sequencing

RNA samples were quality-checked by gel electrophoresis and, when RNA was sequenced, with a Bioanalyzer 2100 device (Agilent Technologies, Santa Clara, USA). Quality-approved samples (RIN between 5.8 and 8.3) were prepared for sequencing with the Illumina TruSeq Stranded Total RNA Sample library kit for plants (Illumina, San Diego, USA). Paired end sequencing (2 × 150 bp) was performed on a NovaSeq6000 machine (Illumina, San Diego, USA) with 54 to 78 million reads per sample.

### RNA sequence quality check

Sequences, both raw and after each filtering step, were quality-checked with FastQC (version 0.11.8; Andrews, 2020) and MultiQC (version 1.7; Ewels *et al.*, 2016). Remaining rRNA sequences were filtered with SortmeRNA (version 2.1; Kopylova *et al.*, 2012) using all available bacterial and eukaryotic rRNA databases with the full search and fastest alignment options. Adapters and low-quality sequences were trimmed with Trimmomatic (version 0.39; Bolger *et al.*, 2014), applying the ILLUMINACLIP function (TruSeq3 paired end adapters) and the SLIDINGWINDOW function (window of 5 bp with a quality score of 20). Sequences shorter than 50 bp were excluded from further analysis.

### Sequence alignment and differential gene expression analysis

Genomic FASTA sequences, cDNA and GTF annotation from *A. thaliana* for mapping the remaining reads were obtained from Ensembl Plants (version TAIR10, release 44; Howe *et al.*, 2020). For read counting, kallisto (version 0.46.0; Bray *et al.*, 2016) with 100 bootstraps was used. Differential gene expression (DGE) analysis was conducted in R (version 3.6.1; R Core Team, 2016) with the ‘DESeq2’ package (Bioconductor version 3.9; Love *et al.*, 2014). R basic syntax was extended with the ‘tidyverse’ package (version 1.3.0; Wickham *et al.*, 2019). Prior to importing the kallisto count files to DESeq2, ‘tximport’ (Bioconductor version 3.9; Soneson *et al.*, 2016) was used to convert count files to the DESeq data format. All genes with a read count >1 were considered valid for further DGE analyses.

### cDNA synthesis and quantitative real-time PCR

First-strand cDNA synthesis was conducted with 2 µg of total RNA in 8 µl reactions with the RevertAid First Strand cDNA Synthesis Kit (ThermoFisher Scientific), following the manufacturer’s protocol. Quantitative real-time PCR (qPCR) was conducted on a CFX96 Touch Real-Time PCR system (Bio-Rad) with 10 µl reactions containing 1 µl cDNA, 0.5 μl of each gene-specific primer (2.5 μM), and 5 μl Power SYBR® Green PCR master mix (Applied Biosystems), using the thermal profile described in Valsamakis *et al.* (2020). The samples were checked for genomic DNA residues with specific primers. We used *AtACT2* (At3g18780), *AtUBQ10* (At4g05320), and *AtGAPDH* (At1g13440) as reference genes (Kozera and Rapacz, 2013). Relative expression levels were calculated according to Livak and Schmittgen (2001). Primers were designed by PerlPrimer (Marshall, 2004) and obtained from Eurofins Genomics (Ebersberg, Germany). A list of the primer sequences is provided in Supplementary Table S1.

We considered as priming marker genes those that were significantly up-regulated in egg-laden, feeding-damaged leaves of 7-week-old plants in the vegetative stage, when compared with egg-free, feeding-damaged genes in leaves of plants of the same developmental stage and age; these genes are *CAX3, PR1, PR2, PR5, PDF1.4* (Lortzing *et al.*, 2019, Valsamakis *et al.*, 2020).

### Larval and seed weight measurements

We measured larval weight as a proxy of plant resistance, as described in Valsamakis *et al.* (2020), and the total weight of seeds as a proxy of plant fitness, using a fine-scale balance (Cubis®, Sartorius Lab Instruments GmbH & Co. KG, Goettingen, DE).

### Hydroponic culture of Arabidopsis

Plants that were assigned to a C/N measurement (see section below), were grown in hydroponic culture (Drechsler *et al.*, 2015). Rockwool was washed in 1% HCl overnight and cleaned again overnight in distilled water. After drying, the rockwool was filled in bottom-cut Eppendorf tubes and autoclaved. Seeds were germinated in rockwool, moistened with distilled water, in darkness and at 22 °C for 1 week. After germination they were kept under SD conditions (8 h/16 h light/dark cycle, 100 µmol m^-2^ s^-1^ light intensity, 21 °C, 40% relative humidity) with distilled water and covered with transparent foil. After 1 week the foil was removed and the distilled water was changed weekly with a hydroponic solution consisting of 0.5 mM KH_2_PO_4_, 0.5 mM MgSO_4_, 0.125 mM K_2_SO_4_, 0.125 mM CaCl_2_, 0.5 mM KNO_3_, 0.005 mM Na-Fe-EDTA, 50 µM KCl, 30 µM H_3_BO_3_, 5 µM MnSO_4_, 1 µM ZnSO_4_, 1 µM CuSO_4_ and 0.7 µM NaMoO_4_ at pH 5.8 with KOH. At the beginning of the fifth week and until the end of the experiment, the hydroponic solution was changed every 3 d with fresh, full-strength growth medium consisting of 1 mM KH_2_PO_4_, 1 mM MgSO_4_, 0.25 mM K_2_SO_4_, 0.25 mM CaCl_2_, 2 mM NH_4_NO_3_, 0.1 mM Na-Fe-EDTA, 50 µM KCl, 30 µM H_3_BO_3_, 5 µM MnSO_4_, 1 µM ZnSO_4_, 1 µM CuSO_4_ and 0.7 µM NaMoO_4_ at pH 5.8 with KOH.

### Determination of carbon (C) and nitrogen (N) content in Arabidopsis

Non-flowering, 7-week-old plants were exposed to *P. brassicae* eggs (E), to *P. brassicae* feeding (F), to both stimuli (EF), or were left untreated as controls (C). After larval feeding for 7 d on the previously egg-laden (EF) or egg-free plants (F), the roots of plants from all treatments were harvested. Roots from egg-laden plants (E) and from untreated control plants (C) were harvested at respective time points. Roots were dried for 5 d at 65 °C. To determine the carbon and nitrogen contents, 2 μg (±0.2 μg) of each sample was encased in a zinc cartridge. The samples were analysed in a Euro EA HEKAtech C/N analyser (HEKAtech GmbH, Wegberg, Germany), using acetanilide (Merck, Darmstadt, Germany) as a standard.

### Statistics and data visualization

Statistical analyses with DESeq2 were performed by assigning ‘treatment’ as the analysis factor in the design formula when comparing non-flowering and flowering plants that had undergone different treatments. The significance level for differentially expressed genes was *P* ≤0.05 after Benjamini-Hochberg correction for multiple testing. Gene ontology (GO) term enrichment analysis was performed on biological processes with DAVID 6.8 (https://david.ncifcrf.gov; Huang *et al.*, 2009). A GO term containing at least three genes was considered enriched at *P*<0.05 after using Fisher’s exact test.

The transcriptome data were visualized with scatterplots after principal component analysis (PCA), heatmaps with hierarchical clustering, and Venn diagrams using the packages ‘pcaexplorer’ (Marini and Binder, 2019), ‘ComplexHeatmap’ (Gu *et al.*, 2016), ‘dentextend’ (Galili, 2015) and ‘eulerr’ (Larson *et al.*, 2018). For hierarchical clustering, we applied Pearson’s correlation and the average linkage methods.

Statistical analyses of larval and plant performance data were conducted with the software R (version 4.0.0; R Core Team, 2016) and R Studio (version 1.2.5042; R Studio Team, 2015). Data were tested for normal distribution with the Shapiro-Wilk test and for homogeneity of variances with Levene’s test. We used the packages ‘car’ (Fox and Weisberg, 2019), ‘lme4’ (Bates *et al.*, 2015), ‘lmtest’ (Hothorn and Zeileis, 2011), ‘multcomp’ (Hothorn *et al.*, 2008), ‘nlme’ (Pinheiro *et al.*, 2022) and ‘psych’ (Revelle, 2017). Statistical details about data analyses are presented in the figure legends and in Supplementary Tables S2, S3.

## Results

### Resistance to herbivory is primed by prior insect egg deposition in non-flowering plants, but not in flowering plants

We assessed plant resistance against larvae feeding on previously egg-laden or egg-free non-flowering and flowering plants by measuring the weights of larvae after feeding. After a larval feeding period of two days, a priming effect of prior egg deposition on larval weights is known to be detectable in non-flowering plants (e.g. Geiselhardt *et al.*, 2013; Lortzing *et al.*, 2019; Valsamakis *et al.*, 2020; Exp. 1-6, Table 1). In Supplementary Table S3 we provide data about larval weights that were determined after the leaf rosettes had been almost completely consumed. We investigated non-flowering and flowering plants of different chronological ages, i.e. the plants were treated at different times after they had been sown. We conducted independent experiments for each plant age.

Larvae that fed on the leaves of previously egg-laden, non-flowering plants gained less weight than larvae on egg-free, non-flowering plants. This egg-mediated priming effect on plant resistance against larvae was found for 5- and 7-week-old, non-flowering plants (Fig. 1A, B; Supplementary Table S3).

The egg-mediated priming effect was absent when larvae fed on the leaves of flowering plants, regardless of the plant’s age, the duration of the feeding period and the photoperiod under which the plants grew (Fig. 1C, D, F; Supplementary Table S3).

Because in nature larvae prefer feeding upon flowers over leaves, and thus move from leaves to flowers (Smallegange *et al.*, 2007), we assessed whether egg-laden leaves systemically prime for greater resistance against larvae that feed on flowers. After 2 d of feeding on the flowers of 12-week-old plants, the larval weights did not appear to be significantly affected by prior egg deposition on the leaves (Fig. 1E).

### Prior egg deposition exerts a much stronger effect on the transcriptome of non-flowering, than flowering, plants

To determine how *P. brassicae* egg deposition, larval feeding, and both together affect transcriptional reprogramming in non-flowering and flowering plants, we used RNA-seq to analyse the transcriptomes of leaves and flowers sampled 24 h after the onset of feeding from 7-week-old non-flowering plants and 12-week-old flowering plants that had grown under SD conditions (Exp. 7, Table 1).

For non-flowering plants, principal component analysis (PCA) revealed distinctive differences between the transcriptomes of feeding-damaged leaves (F and EF) and the transcriptomes of non-damaged leaves (C and E), explaining 55.60% of the total variance, as indicated by PC1. PC2 explained 13.17% of the total variance and could separate the transcriptomes of egg-laden (E and EF) from egg-free leaves of these plants (C and F; Fig. 2A, left panel).

For flowering plants, the transcriptomes of feeding-damaged leaves with or without eggs (F and EF) were able to be separated from the transcriptomes of leaves from non-damaged plants (C and E), explaining 33.36% of the total variance by PC1 (Fig. 2A, central panel). The transcriptomes of egg-laden leaves from flowering plants (E and EF) were not distinguishable from transcriptomes of egg-free flowering plants (C and F). Furthermore, the transcriptomes of feeding-damaged, egg-free leaves of flowering plants were not distinguishable from those of feeding-damaged, egg-laden ones (F versus EF). There were no obvious differences when comparing the transcriptomes of undamaged, egg-laden, and undamaged, egg-free leaves of flowering plants (E versus C; PC2). The PCA of flower transcriptomes of the differently treated plants were not distinguishable from one another (Fig 2A, right panel).

Insect egg deposition resulted in different numbers of differentially expressed genes in leaves from non-flowering and flowering plants (Fig. 2B). While non-flowering plants showed more than 1000 differentially expressed genes in response to eggs, fewer than 100 genes were differentially expressed in egg-laden flowering plants.

In non-flowering plants, numerous genes responded to the egg deposition. When comparing transcriptomes of egg-laden leaves with those of untreated leaves, the expression of 1292 genes was up-regulated and expression of 614 genes was down-regulated (Fig. 2B, left panel, E versus C, Supplementary Dataset S1). GO term analysis of the up-regulated genes revealed that terms related to defence responses (e.g. to bacteria, fungi, or wounding), immune responses [including hypersensitive responses and systemic acquired resistance (SAR)] and to responses to phytohormones [including SA, jasmonic acid (JA) and abscisic acid (ABA)] were overrepresented (Supplementary Dataset S2). Significantly enriched GO terms of down-regulated genes were related to auxin- and brassinosteroid-mediated signalling (Supplementary Dataset S2).

In the leaves of flowering plants, 77 genes were up-regulated, and nine were down-regulated, in response to eggs (Fig. 2B, central panel, E versus C). Here, most counts of the up-regulated genes were grouped according to GO terms relating to oxidative stress responses, whereas analysis of the down-regulated genes did not reveal a significant enrichment of GO terms (Supplementary Dataset S2).

Larval feeding affected the expression of a large number of genes. When comparing the transcriptomes of egg-free, feeding-damaged leaves and untreated control leaves of non-flowering plants, 5871 genes (3342 up-regulated and 2529 down-regulated) were differentially expressed (Fig. 2B, left panel, F versus C, Supplementary Dataset S1). Among the up-regulated genes, GO terms related to responses to wounding, bacteria and fungi, and to the phytohormones JA, SA, and ABA, were significantly enriched. Among the down-regulated genes, the GO terms ‘photosynthesis’ and ‘abiotic stress responses’ were significantly enriched (Supplementary Dataset S2).

In contrast to the transcriptional response of non-flowering plants to larval feeding, fewer than half as many genes were regulated in flowering plants (1639 up-regulated and 977 down-regulated) in response to feeding (Fig. 2B, central panel, F versus C, Supplementary Dataset S1). The functions of these genes in flowering plants show similarities to the functions of differentially expressed genes in the feeding-damaged leaves of non-flowering plants. For example, among the up-regulated genes, GO terms related to defence responses towards biotic stresses and to the JA, SA, and ABA pathways, and among the down-regulated genes, GO-terms related to abiotic stresses were similarly enriched (Supplementary Dataset S2).

Remarkably, prior egg deposition strongly affected the transcriptome of feeding-damaged leaves from non-flowering plants, but not the transcriptome of feeding-damaged leaves from flowering plants (Fig. 2B, EF versus F).

In non-flowering plants, 3145 genes (1626 up-regulated and 1519 down-regulated) were differentially regulated compared with feeding-damaged leaves with and without prior egg deposition (Fig. 2B, left panel, EF versus F). The significantly enriched GO terms of up-regulated genes are related to SAR, leaf senescence, and SA biosynthesis and signalling (Supplementary Dataset S2). Among the genes were *SALICYLIC ACID INDUCTION DEFICIENT 2* (*SID2*, At1g74710), *PHYTOALEXIN DEFICIENT 4* (*PAD4*, At3g52430), *PR1* (At2g14610), *PR2* (*BGL2*, At3g57260), *PR5* (At1g75040) and *AGD2-LIKE DEFENSE RESPONSE PROTEIN 1* (*ALD1,* At2g13810). Many of the up-regulated genes play a role in ABA-mediated signalling [e.g. *2-DOMAIN ABA-RELATED8* (*CAR8*, At1g23140), *GLUTATHIONE PEROXIDASE 3* (*GPX3*, At2g43350), *CYSTEINE-RICH RECEPTOR-LIKE PROTEIN KINASE 45* (*CRK45*, At4g11890)], and nine genes are involved in JA-mediated signalling [e.g. *SYNTAXIN OF PLANTS 121* and *122* (*SYP121*, At3G11820 and *SYP122*, At3G52400), *RING DOMAIN LIGASE 4* (*RGLG4*, At1g79380); Supplementary Datasets 1, 2, EF versus F vegetative]. Furthermore, three GO terms related to calcium homeostasis and transport were significantly enriched. Among the up-regulated genes involved in calcium-mediated signalling are the cation/proton exchanger genes *CAX3* (At3g51860) and *CAX5* (At1g55730), seven *CPK*s (calcium-dependent protein kinases), eight *GLR*s (amino acid gated calcium channels) and seven *CNGC*s (cyclic nucleotide gate channels; Supplementary Datasets 1, 2). Significantly enriched GO terms of down-regulated genes are related to photosynthesis, carbohydrate metabolic processes and JA biosynthesis and signalling, the latter including genes such as *OXOPHYTODIENOATE-REDUCTASE 3* (*OPR3*, At2g06050), *JASMONATE INSENSITIVE 1* (*MYC2*, At1g32640) and *JASMONATE-ZIM-DOMAIN PROTEIN 9* (*JAZ9*, At1g70700; Supplementary Dataset S1, EF versus F vegetative down).

In flowering plants, the comparison of feeding-induced transcriptomes of previously egg-laden leaves and egg-free leaves revealed only five differentially up-regulated genes and 17 down-regulated genes (Fig. 2B, middle panel, EF versus F). The up-regulated genes included *OSMOTIN 34* (*OSM34*, At4g11650) and *BASIC CHITINASE* (*CHI-B*, At3g12500); the latter is a basic chitinase involved in SAR. The down-regulated genes included six chloroplast-encoded ribosomal proteins (*RPS*s; Supplementary Dataset S1, EF versus F, reproductive, leaf).

The transcriptomes of flowers were not affected by any of the treatments (Fig. 2B, right panel). Only one gene was down-regulated in response to egg deposition and subsequent larval feeding (Supplementary Dataset S1). Hence, the treatment of leaves had almost no systemic effect on the flower transcriptome.

### The transcriptional response of non-flowering plants to insect eggs overlaps with the response to larval feeding damage

To gain deeper insight into the specific mechanism by which the transcriptome of non-flowering plants responds to egg deposition and larval feeding, we compared the transcriptional responses of leaves from non-flowering plants exposed to eggs, to larval feeding, or to both stimuli (Fig. 3).

The transcriptional response of non-flowering plants to insect eggs shows some overlap with that to larval feeding (E versus C, and F versus C). Of the genes differentially up-regulated in response to eggs, 784 (61%) were also up-regulated in response to larval feeding. Of the genes down-regulated in ­response to the egg treatment, 341 (56%) were also down-regulated in response to feeding (Fig. 3; Supplementary Dataset S3). Significantly enriched GO terms of the up-regulated genes are related to defence responses to several organisms, SAR and responses to SA, JA and ABA. Among the up-regulated genes, marker genes related to priming responses such as *PR5, PR2, PDF1.*4 and *CAX3*, were commonly induced by eggs or larval feeding, often to similar extents (Supplementary Dataset S3). The enriched GO terms of down-regulated genes are related to auxin, cytokinin and brassinosteroid responses (Supplementary Dataset S4).

The plant’s transcriptional priming response (EF versus F) showed 869 (53%) up-regulated and 623 (41%) down-regulated genes that were also regulated either (i) in response to eggs (E versus C), (ii) in response to larval feeding (F versus C), or (iii) in response to eggs and to larval feeding (E versus C, and F versus C; Fig. 3, grey intersections, Supplementary Dataset S3). The significantly enriched GO terms of commonly up-regulated genes are, among others, related to responses to bacteria, fungi and wounding, SAR, and SA- and ABA-mediated signalling, whereas the GO terms of the down-regulated genes are related to circadian rhythm, meristem development and trehalose biosynthesis (Supplementary Dataset S4).

### The egg-primed, feeding-induced transcriptome of non-flowering plants shows substantial overlap with the transcriptome of untreated flowering plants

Given that in Exp. 2 the flowering plants were 5 weeks older than non-flowering plants, we addressed the question as to what degree the transcriptomes of these untreated plants differ. In total, 7140 genes (3698 up-regulated and 3442 down-regulated) were differentially expressed when comparing untreated, flowering plants with untreated, non-flowering ones (Supplementary Dataset S1, Crep versus Cveg). As expected, these data show a massive transcriptional reprogramming in plants after their transition from the vegetative to the reproductive stage.

We then focussed on the expression of priming-responsive genes, i.e. genes that were differentially expressed in non-flowering, feeding-damaged plants with prior egg deposition, compared with feeding-damaged plants without prior egg deposition (EF versus F, vegetative, Supplementary Dataset S1). We compared the expression pattern of these genes with those in flowering, feeding-damaged plants with and without prior egg deposition (EF versus F, reproductive) and in untreated flowering plants relative to non-flowering plants (Crep vs. Cveg; Fig. 4A).

The transcriptional priming response to feeding damage that we found in egg-laden, non-flowering plants almost completely vanished in egg-laden, flowering plants (EF versus F, vegetative compared with EF versus F, reproductive).

The expression pattern of priming-responsive genes in non-flowering plants (EF versus F, vegetative) was similar to that detected when we compared untreated flowering plants with untreated non-flowering plants (Crep versus Cveg). The egg-primed, feeding-induced transcriptome (EF versus F) of non-flowering plants shared 1460 (47%) genes (729 up-regulated and 731 down-regulated genes; Fig. 4B) with the differentially regulated transcriptome of control leaves from flowering plants (Crep versus Cveg; Supplementary Dataset S5). Of these, genes with GO terms associated with defence responses, senescence, cell death, and SAR- or SA-mediated signalling, among others, were significantly enriched (Supplementary Dataset S6).

### The egg-primed anti-herbivore response depends on the plant’s ontogenetic but not chronological age

To disentangle whether egg-priming of herbivore resistance is dependent on a plant’s ontogenetic stage or on its chronological age, we analysed the larval weights on egg-laden or egg-free flowering and non-flowering plants of the same chronological age (Exp. 8 and 9, Table 1).

On non-flowering, 11-week-old, egg-laden plants, larvae gained less weight than on egg-free, non-flowering plants of an equivalent age. This priming effect indicated by larval weight was absent in the flowering 11-week-old plants (Fig. 5; Exp. 8, Table 1). Interestingly, larvae that fed on flowering plants gained significantly less weight than larvae on non-flowering plants (*P*=2.7 × 10^-07^; Fig. 5).

A comparative qPCR analysis of priming marker genes in 11-week-old untreated flowering, and non-flowering, plants showed a much higher constitutive expression level of these genes in the untreated flowering plants (Fig. 6). These data are in accordance with the RNA-seq data that showed constitutively enhanced expression of priming-responsive genes in leaves from untreated flowering plants compared with untreated non-flowering plants (Fig. 4).

We also studied larval weights on 7-week-old flowering and non-flowering WT plants and on 7-week-old *svp-32* mutant plants that show an early flowering phenotype under SD (Wilson *et al.*, 2017; Exp. 9, Table 1). On non-flowering, egg-laden WT plants, larvae gained less weight than on egg-free plants, whereas no such priming effect on larval weight was detectable in flowering plants (Fig. 7). Again, the larvae gained less weight on flowering WT plants than on non-flowering plants, but this effect was only visible after 6 d, rather than 2 d, of feeding (Fig. 7). No priming effect on larval weight was found in egg-laden, flowering *svp-32* mutant plants (Fig. 7).

A qPCR analysis of priming marker genes in 7-week-old non-flowering plants showed that the expression levels of these genes were significantly higher in previously egg-laden leaves than in egg-free leaves, thus confirming our previous results (Lortzing *et al.*, 2019, Valsamakis *et al.*, 2020). However, with the exception of *PR1,* expression of these genes was not higher in EF than F leaves of flowering plants. Instead, the genes showed constitutively high expression levels in the untreated control leaves of flowering plants. After 2 d of feeding, the expression levels of the analysed genes in both egg-free and egg-laden flowering (F, EF) plants were about as high as the expression levels in EF leaves of non-flowering plants (Fig. 8).

Our analysis of the expression levels of primable marker genes in the flowering *svp-32* mutant showed that prior egg deposition did not significantly enhance expression of primable, feeding-induced genes (Fig. 8; *P*-values are provided in Supplementary Table S2).

Additionally, we analysed the expression of the JA-responsive genes *MYC2*, *VSP1* (*VEGETATIVE STORAGE PROTEIN 1*) and *PR4* (*PATHOGENESIS-RELATED* 4) in 7- and 11-week-old flowering and non-flowering plants (Figs 6, 8). In 7-week-old flowering and non-flowering WT and *svp-32* plants, the expression of these genes was induced by larval feeding (Fig. 8). However, prior egg deposition did not further enhance their expression, but rather attenuated it (Fig. 8, *MYC2* and *VSP1*). *PR4* was constitutively more highly expressed in flowering than in non-flowering plants, regardless of the plant’s age (Figs 6, 8).

In summary, these results provide further evidence that egg-mediated priming of herbivore resistance is dependent on a plant’s ontogenetic stage, but not on its chronological age.

### Non-flowering egg-primed plants produce a higher seed weight than non-primed plants after recovery from feeding damage

To determine the possible costs of responses to eggs and feeding damage, we measured the total seed weight per plant (Fig. 9A, Exp. 10; Table 1). Plants exposed in the vegetative stage (7-week-old) to larval feeding damage did not fully compensate for the tissue lost to herbivory. They showed a reduced total seed weight after regrowth. However, the plant’s response to eggs mitigated the loss in total seed weight due to feeding damage. Plants treated in the vegetative stage with egg deposition and subsequent larval feeding produced a higher total seed weight after regrowth than egg-free plants damaged by feeding. In our analyses, while the factor ‘feeding’ significantly affected the total seed weight of regrown plants (*P*=2.7 × 10^-4^), ‘eggs’ had no significant impact on seed weight (*P*=0.17). However, our data show a clear interaction effect between ‘eggs’ and ‘larval feeding’ (*P*=0.03; Fig. 9A). Untreated C-plants that were transferred to abiotic conditions to allow flowering at the age of 7 weeks produced less seed weight than C-plants that were transferred to flowering conditions at the age of 12 weeks. This might be due to the experimental conditions, in which the 12-week-old plants had been sown 5 weeks earlier than the 7-week-old plants; thus the 12-week-old plants flowered for longer under SD conditions than did 7-week-old plants, before all plants were simultaneously transferred to LD conditions for recovery after feeding damage (Fig. 9A). Supplementary Fig. S1 shows similar results obtained by a repeated independent experiment in the greenhouse.

In contrast, regrown plants that had been treated during the reproductive stage with insect eggs and/or larval feeding on their leaves produced as much seed weight as untreated flowering plants (Fig. 9A). Hence, the total seed production of these plants was unaffected by both the insect egg deposition and larval feeding.

We addressed the question of whether the higher seed production in regrown plants exposed to egg deposition and feeding damage during the vegetative stage was due to an egg-mediated change in resource allocation to the roots of feeding-damaged plants, thus facilitating later regrowth and seed set (Exp. 11, Table 1). However, the quantification of C and N concentrations in the roots did not show any indication of a resource allocation into the roots of non-flowering plants in response to any treatment (Table 2).

Instead, higher seed production was seen in EF than in F plants that had been treated during the vegetative stage; this might be due to more abundant aboveground resources and/or increased photosynthesis in EF plants, which have more leaf tissue remaining after feeding than F plants (Geiselhardt *et al.*, 2013). We tested this hypothesis by cutting off all the remaining leaf tissue on non-flowering EF and F plants at the end of the feeding period (Exp. 12, Table 1). In support of this hypothesis, the plants produced similar seed weights after regrowth (Fig. 9B).

## Discussion

Our study shows that resistance of Arabidopsis against insect feeding is primable by prior insect egg deposition when the plant is in its vegetative stage, but not in its reproductive stage. It is the ontogenetic stage of the plant that appears to be decisive for this effect, not its chronological age. Plants of the same chronological age were only primable by prior egg deposition when they were in the vegetative stage, but no priming effect was detectable once the plants had reached the reproductive stage. Larvae performed equally well on egg-free and previously egg-laden flowering plants, but performed worse on previously egg-laden, non-flowering plants than on egg-free non-flowering plants. When studying the costs of the plant’s primability, we found that non-flowering plants produce a lower total seed weight after recovery from herbivory, but benefit from the egg-priming, which mitigates this loss. In contrast, the total seed weight of flowering plants that had been exposed to eggs and/or larval feeding was comparable after regrowth to that of the seed weight of untreated plants. These results suggest that Arabidopsis plants in the reproductive stage tolerate *P. brassicae* larval herbivory. Our finding that the defence of flowering plants against herbivory is not primable by prior egg deposition is in accordance with our transcriptomic studies. In contrast to the massive transcriptional reprograming in non-flowering plants in response to insect eggs, flowering plants showed very few transcriptional responses to eggs. However, their transcriptomes showed constitutively high expression of defence-related priming genes.

### Which factors and mechanisms contribute to the loss of egg-mediated primability of resistance against herbivory in flowering Arabidopsis?

The reduced responsiveness of flowering Arabidopsis to insect egg deposition is indicated by there being very few differentially regulated genes in response to egg deposition, while feeding damage induced a significant transcriptomic reprogramming comparable to that in non-flowering plants (Fig. 4B). Accordingly, the transcriptomic responses of flowering plants to insect eggs followed by feeding damage hardly differed from the response to feeding damage alone (Fig. 2). Almost half of the primable genes (i.e. those which are differentially regulated in non-flowering EF plants when compared with F plants) were also differentially regulated in untreated flowering plants when compared with untreated non-flowering plants (Crep versus Cveg; Fig. 4B). These primable genes play a role in SA-mediated signalling and defence responses in particular (Supplementary Dataset S6).

In non-flowering plants, *P. brassicae* egg deposition alone, or larval feeding alone, increases SA levels and expression of SA-responsive genes, including priming markers *PR1, PR2* and *PR5, CAX3* and *PDF1.4* (e.g. Little *et al.*, 2007; Bruessow *et al.*, 2010; Lortzing *et al.*, 2020; Valsamakis *et al.* 2020). This similar response to eggs and feeding contributes to the priming response mechanism in an additive or synergistic manner when plants are exposed to both stimuli, resulting in accelerated resistance responses to the larvae (Fig. 3; Altmann *et al.*, 2018; Lortzing *et al*., 2019, 2020; Valsamakis *et al.*, 2020).

While non-flowering plants accumulate SA and express SA-related genes in response to insect eggs, untreated plants do so when switching from the vegetative to the reproductive stage and start flowering (e.g. Rivas-San Vicente and Plasencia, 2011; Carella *et al.*, 2015; Kazan and Lyons, 2016). Accordingly, flowering plants show constitutively high expression of priming marker genes. However, neither egg deposition nor larval feeding further enhanced the expression of these genes (Figs 2, 6, 8). Because the SA level and SA-responsive traits are known to be high in flowering plants (Kazan and Lyons, 2016), we suggest that insect egg depositions do not have sufficient inductive capacity to further enhance SA-related traits.

The reduced responsiveness of flowering plants to *P. brassicae* eggs and the lack of a priming response may also be due to increasing thickness and toughness of its leaves during development. A thick leaf cuticle and tough epidermal cell wall may reduce diffusion and perception of phosphatidylcholine derivatives, which are associated with *P. brassicae* eggs and elicit plant responses, including enhanced expression of *PR1* (Stahl *et al.*, 2020), a typical priming marker gene (Valsamakis *et al.*, 2020). However, the early flowering *svp*-*32* mutant has very thin leaves, and its response to larvae was not primable by prior egg deposition (Fig. 7). Thus, it is unlikely that flowering plants are less sensitive to *P. brassicae* eggs because of increased leaf thickness.

### Which factors contribute to the poor performance of *Pieris brassicae* larvae on flowering plants?

In our study, larvae gained less weight on flowering plants than on egg-free, non-flowering ones, regardless of whether the flowering plants had received eggs or not prior to larval feeding. This worse performance of larvae on flowering plants might be due to a lower nutritional quality of the leaves, which usually become senescent when plants enter the reproductive stage and start exporting nutrients from leaves to developing reproductive organs (Kim *et al.*, 2018).

Alternatively, the worse larval performance on flowering plants might be due to constitutively higher resistance traits in this developmental stage. Amongst others, ABA, ethylene, JA, and SA are positive regulators of senescence, and their concentrations increase when leaves start aging (Kim *et al*., 2015, 2018; Xu *et al.*, 2020; Zhang *et al.*, 2020). Our transcriptional data show that ABA-, ethylene-, JA- and SA-responsive genes are constitutively expressed at high levels in leaves of flowering plants (Figs 6, 8; Supplementary Dataset S1, S2).

The high expression levels of JA- and ABA-responsive genes in flowering plants may result in enhanced biosynthesis of defensive compounds that harm the larvae. However, *P. brassicae* larval weight was not affected when larvae fed upon a *coi1* mutant, which shows reduced sensitivity to JA (Schweizer *et al.*, 2013). As a specialist herbivore on Brassicaceae, *P. brassicae* larvae can deal with some JA-inducible, brassicaceous-characteristic plant defence compounds such as glucosinolates; nitrile specifier proteins in the larval gut divert the hydrolysis of glucosinolates toward the formation of less toxic nitriles instead of the highly toxic isothiocyanates (Wittstock *et al.*, 2004).

In addition to JA- and ABA-responsive genes, SA-responsive genes were constitutively highly expressed in flowering plants. Among those genes are priming markers (e.g. *PR1, PR2, PR5, CAX3, PDF1.4, ALD1;*Figs 6, 8), which have been shown to be crucial for the egg-mediated priming of feeding-induced resistance in non-flowering plants (Lortzing *et al.*, 2019). These results suggest that defences of Arabidopsis against *P. brassicae* larvae are regulated by SA signalling. Future studies need to test this hypothesis, and to elucidate the contribution of the different phytohormone pathways to the resistance traits of flowering Arabidopsis against *P. brassicae* larval feeding. We suggest that more than one phytohormone pathway contributes to plant resistance against *P. brassicae*.

### Is the high resistance to herbivory of flowering plants dependent on their chronological age or their developmental stage?

Greater resistance of Arabidopsis with increasing chronological age has been demonstrated against two Lepidopteran species, the generalist *Helicoverpa armigera*, and the specialist for brassicaceous plants, *Plutella xylostella*. Both insect species gain less weight on adult plants than on juvenile plants, although the JA-mediated response declines with advancing plant chronological age (Mao *et al.*, 2017). However, our data show that a high herbivore resistance coincides with the plant’s flowering stage, but not with the plant’s chronological age (Figs 5, 7B).

These findings also suggest that herbivore resistance in *A. thaliana* has no direct parallel with so-called ‘age-related resistance’ (ARR) against (hemi-)biotrophic pathogens (Hu and Yang, 2019). ARR against pathogens co-occurs with the transition to flowering, but does not require flowering. The early flowering mutant *svp* is ARR-defective against pathogens (Wilson *et al.*, 2017). In our study, 7-week-old flowering *svp-32* plants showed no egg-primability of herbivore resistance, but did show—independent of prior egg deposition—a strong resistance against herbivory (Fig. 7).

To obtain WT plants of the same chronological age, but in different developmental stages (Figs 5, 7), we exposed them to different photoperiods. Therefore, we cannot entirely discount the possibility that differing photoperiods, rather than different developmental stages of the WT plants, might have affected their resistance by hindering the herbivore’s performance. However, before the plants in different developmental stages were treated with larvae, they grew under the same light conditions for almost 2 weeks (1 week acclimatization and 6 d prior to their treatment with larvae), thus reducing the likelihood of an effect caused by the photoperiod. Furthermore, the flowering *svp-32* mutant plants that grew under SD similar to non-flowering WT, showed constitutively greater resistance than non-flowering WT plants.

### Which factors mitigate the loss in seed weight of egg-primed plants after their recovery from feeding damage during the non-flowering stage?

Since leaf damage may induce resource allocation to the roots—as shown, for instance, by Ferrieri *et al.* (2013) – we tested the hypothesis that prior egg deposition enhances resource allocation to roots, thus making possible an improved seed set when regrowing. However, we did not find evidence to support this hypothesis in our measurements of C and N concentrations in the roots of non-flowering plants exposed to eggs, larval feeding, or to both (Table 2).

Alternatively, the mitigated seed loss in egg-primed plants after feeding damage during the non-flowering stage might be due to higher photosynthetic activity in these plants. This hypothesis is based on our previous study which showed less feeding damage in egg-primed, non-flowering plants than in egg-free plants (Geiselhardt *et al.*, 2013). Since egg-primed, non-flowering plants suffer less larval feeding damage, they have more leaf tissue remaining for photosynthesis and thus might have more energy resources than non-primed, feeding-damaged plants. This hypothesis is supported by data presented in Fig. 9B. When all residual leaf tissue was cut away after the larval feeding period, previously egg-laden and egg-free plants produced the same seed weight after their recovery.

### What are the ecological consequences of the loss of egg-primable resistance in flowering Arabidopsis?

With regards to seed production, our data showed that flowering *A. thaliana* can tolerate *P. brassicae* larval herbivory. When flowering plants were fed upon by *P. brassicae,* the regrown plants produced as much seed weight as plants that had not been fed upon, independent of prior egg deposition on either plant (Fig. 9A). Previous studies have already shown that flowering Arabidopsis plants are more tolerant of herbivory or artificial damage than non-flowering plants (Tucker and Avila-Sakar, 2010; Akiyama and Ågren, 2012).

While the non-flowering Arabidopsis plants tested in our study suffered from herbivory and produced less seed weight after regrowth, a field study by Pashalidou *et al.* (2015b) showed that egg-free *Brassica nigra* plants exposed to *P. brassicae* larval herbivory during their vegetative stage tolerate herbivory as well as, and produce a number of seeds after regrowth comparable to, untreated control plants. Prior egg deposition on non-flowering, feeding-damaged *B. nigra* even significantly increased the number of seeds produced by regrown plants, suggesting a priming of tolerance (Pashalidou *et al.*, 2015b). This overcompensation and priming of tolerance might be explained by a wide range of favourable environmental conditions (Agrawal, 2000). As with our study, another brassicaceous plant, *Raphanus sativus*, has been shown to be more tolerant to damage during its reproductive stage, but less resistant than during its vegetative stage (Boege *et al.*, 2007). The plant strategy ‘tolerance’ as a means of coping with herbivory might be preferred over ‘resistance’ by some plant species when they reach the reproductive stage (Stowe *et al.*, 2000; Boege and Marquis, 2005).

However, our results suggest that in addition to tolerance, flowering Arabidopsis are more resistant to *P. brassicae* larvae than egg-free, non-flowering plants, as discussed above.

### Does egg deposition on leaves systemically prime resistance of flowers against florivory?

Systemic signalling in response to *P. brassicae* eggs has been reported for Arabidopsis from local to distal leaves (Hilfiker *et al.*, 2014; Alfonso *et al.*, 2021), and systemic changes in flowers have been reported in response to leaf herbivory by *P. brassicae* on flowering *B. nigra* plants in the form of an emission of floral odour (Bruinsma *et al.*, 2014). However, our results show that Arabidopsis flowers were not primed by egg deposition on the leaves; in fact, the performance of larvae feeding on the flowers of plants that had experienced eggs on their leaves was unaffected (Fig. 1E). This ecological result was also reflected at the transcriptional level, where the transcriptome of flowers did not appear to change in response to eggs, nor to larval feeding (Fig. 2).

In summary, the primability of responses to insect herbivory in Arabidopsis changes during ontogeny. While plants in the vegetative stage are responsive to insect eggs, which are taken as a cue to prime for improved resistance against hatching larvae, plants in the reproductive stage show little response to insect eggs, instead appearing to be more resistant against larvae and tolerant of herbivory without a loss of reproductive fitness. This loss of primability in plants depends on their developmental stage, but not on their chronological age.

From a mechanistic point of view, egg deposition induces SA-related defence traits in non-flowering plants (e.g. Bruessow *et al.*, 2010; Lortzing *et al.* 2019), whereas SA-related traits are constitutively more highly expressed in flowering plants; egg deposition cannot further induce these traits in plants at this reproductive stage. Our study suggests that plants in the reproductive stage can ‘afford’ a weaker responsiveness to insect eggs and the resulting loss of primability, because—even in the absence of any stimulus—their transcriptome is already in a state that partially matches that of egg-primed, feeding-induced leaves from non-flowering plants. This could also explain the poorer performance of larvae on egg-free, flowering plants compared with the larvae on egg-free, non-flowering plants. Our data show that the defences of plants in the reproductive stage are associated with a differential expression of many of those genes, which are egg-primable in feeding-induced, non-flowering plants, but differentially regulated in the flowering plants, independent of the treatment. Hence, our data suggest that transcriptional reprogramming during Arabidopsis ontogeny results in a shift from primable resistance against herbivory to non-primable tolerance and greater non-primable constitutive resistance.

As other natural Brassicaceae host plant species of *P. brassicae* in the vegetative stage show improved defences against larvae when they have been primed by previous ovipositioning (Pashalidou *et al.*, 2015a), future studies need to investigate whether these plant species lose the primability of their resistance against herbivory after having entered the reproductive stage, as is the case with Arabidopsis.

## Supplementary data

The following supplementary data are available at *JXB* online.

Table S1: List of qPCR primers.

Table S2: Statistical details of the data analysed for Exps 8-12.

Table S3: Weights of *Pieris brassicae* larvae on egg-laden or egg-free non-flowering and flowering plants. Additional data and statistical details, Exps 1-6.

Fig. S1. Impact of *Pieris brassicae* egg deposition and larval feeding damage on seed production of subsequently regrown Arabidopsis plants in the vegetative stage; Exp. 10.

Dataset S1. List of differentially expressed genes in Arabidopsis treated in the vegetative or reproductive stage with *Pieris brassicae* eggs, larval feeding, or both eggs and larval feeding; Exp. 7.

Dataset S2. Biological process GO term enrichment of differentially expressed genes in Arabidopsis treated in its vegetative or reproductive stage with *Pieris brassicae* eggs, larval feeding, or both eggs and larval feeding; Exp. 7.

Dataset S3. List of differentially regulated genes in Arabidopsis leaves treated during the vegetative stage; Exp. 7.

Dataset S4. Biological process GO term enrichment of genes commonly regulated in response to eggs, to feeding, and to both stimuli in Arabidopsis leaves treated during the vegetative stage; Exp. 7.

Dataset S5. List of commonly and uniquely regulated genes in egg-laden versus egg-free, non-flowering Arabidopsis leaves after larval feeding damage, compared with differentially expressed genes in untreated control leaves from flowering versus non-flowering plants; Exp. 7.

Dataset S6. Biological process GO term enrichment of egg-primable genes in non-flowering Arabidopsis and of differentially expressed genes when comparing untreated control leaves of flowering and non-flowering plants; Exp. 7.

erac199_suppl_supplementary_dataset_S1

erac199_suppl_supplementary_dataset_S2

erac199_suppl_supplementary_dataset_S3

erac199_suppl_supplementary_dataset_S4

erac199_suppl_supplementary_dataset_S5

erac199_suppl_supplementary_dataset_S6

erac199_suppl_supplementary_figure_S1_tables_S1-S3

## Data Availability

RNA-seq raw sequence data were deposited at the European Bioinformatics Institute (EBI) platforms ArrayExpress and Expression Atlas under the accession number E-MTAB-9196 (https://www.ebi.ac.uk/arrayexpress/experiments/E-MTAB-9196/). Further data are included in the article or its supplementary data.
