# Supplementary material for: Priming of Arabidopsis resistance to herbivory by insect egg deposition depends on the plant’s developmental stage
Source: J Exp Bot. 2022 May 6;73(14):4996–5015. doi: 10.1093/jxb/erac199 (PMC9366327; doi:10.1093/jxb/erac199)
Supplement: erac199_suppl_supplementary_figure_S1_tables_S1-S3 [file erac199_suppl_supplementary_figure_s1_tables_s1-s3.pdf]

Priming of Arabidopsis resistance to herbivory by insect egg deposition depends on the plant's developmental stage

Georgios Valsamakis[1], Norbert Bittner[2], Reinhard Kunze[2], Monika Hilker[1] and Vivien Lortzing[1]*

Overview

Supplementary Figures:

Supplementary Fig. S1 Impact of *Pieris brassicae* egg deposition and larval feeding damage on seed production of later regrown Arabidopsis plants. Treatments of plants in their vegetative stage, seven weeks old (experiment 10).

Supplementary Tables:

Supplementary Table S1: List of qPCR primers.

Supplementary Table S2: Statistical details of analysed data of experiments 8-12.

Supplementary Table S3: Weights of *Pieris brassicae* larvae on egg-laden or egg-free non-flowering and flowering plants: Additional data and statistical details, experiments 1-6.

Supplementary Datasets:

Supplementary Data 1 List of differentially expressed genes in Arabidopsis treated in the plant's vegetative or reproductive stage with *Pieris brassicae* eggs, larval feeding or eggs and larval feeding; experiment 7.

Supplementary Data 2 Biological process GO-term enrichment of differentially expressed genes in Arabidopsis treated in the plant's vegetative or reproductive stage with *Pieris brassicae* eggs, larval feeding or eggs and larval feeding; experiment 7.

Supplementary Data 3 List of differentially regulated genes in Arabidopsis leaves treated in the vegetative stage; experiment 7.

Supplementary Data 4 Biological process GO-term enrichment of genes commonly regulated in response to eggs, to feeding and to both stimuli in Arabidopsis leaves treated in the vegetative stage; experiment 7.

Supplementary Data 5 List of commonly and uniquely regulated genes in egg-laden vs egg-free non-flowering Arabidopsis leaves after larval feeding damage compared to differentially expressed genes in untreated control leaves from flowering versus non-flowering plants; experiment 7.

Supplementary Data 6 Biological process GO-term enrichment of egg-primable genes in non-flowering Arabidopsis and of differentially expressed genes when comparing untreated control leaves of flowering and non-flowering plants, experiment 7.

Supplementary Figure

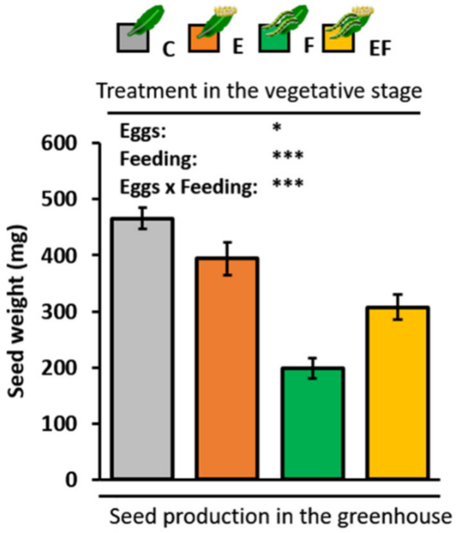

Fig. S1 Impact of *Pieris brassicae* egg deposition and larval feeding damage on seed production of later regrown *Arabidopsis thaliana* plants (compare Table 1, Exp. 10). Total seed weight in mg (means ± SE) produced by *A. thaliana* plants treated in the vegetative stage (seven-week-old) with *P. brassicae* eggs (E, orange bars), feeding by *P. brassicae* larvae (F, green bars) or eggs followed by feeding (EF, yellow bars), or they were left untreated as controls (C, grey bars). After plants recovered, they were transferred to the greenhouse to produce seeds. Statistical differences among the treatments were evaluated with a linear mixed model with eggs, feeding and their interaction as fixed factors and experimental block as random factor. The asterisks represent significant effects of the factors at the level *** $P < 0.001$, * $P < 0.05$. The number of replicates (plants) is $N = 10 – 12$ (plants) per treatment. Statistical details are listed in Table S2.

Supplementary Tables

Supplementary Table S1: List of qPCR primers.

Target	AGI	Sequence 5´→3´ Forward	Reverse
ACT2	AT3G18780	CTTCCCTCAGCACATTCCAG	GACCTGCCTCATCATACTCG
UBQ10	AT4G05320	GGCCTTGTATAATCCCTGATGAATAAG	AAAGAGATAACAGGAACGGAAACATAGT
GAPDH	AT1G13440	TTGGTGACAACAGGTCAAGCA	AAACTTGTCGCTCAATGCAATC
genomic control		TTTTTTGCCCCCTTCGAATC	ATCTTCCGCCACCACATTGTAC
PR1	AT2G14610	ACACGTGCAATGGAGTTTGTGG	TTGGCACATCCGAGTCTCACTG
PR2	AT3G57260	CACAGCTGGACAAATCGGAG	CAAGATCTGAACTGGGAACGTC
PR5	AT1G75040	GATGTGAGCCTCGTAGATGGT	ACATTGTTCTGATCCATGACCT
CAX3	AT3G51860	CTTTCTACACTGGTCCAACAGTG	TATTTCACCACTGCCACTTTGTTA
PDF1.4	AT1G19610	ACACACTTATGCTCTTCCTTTGCC	ACCGCCATCATCTCAGTGGAAG
MYC2	AT1G32640	AACCACGTCGAAGCAGAGAGAC	TTGGTACAACCGCTCGTAACGC
PR4	AT3G04720	CGCCACCTACCATTTCTATAATCC	CTTGTGTTCTTCACCCTTAAACAC
VSP1	AT5G24780	ATCTCATACTCAAGCCAAACGG	TCCTCAACCAAATCAGCCCA

Supplementary Table S2: Statistical details of analysed data of experiments 8-12 (except larval performance studies, for which details are provided in Table S3). Exp no.: Experiment number (overview in Table 1, main text)

Exp no.	Statistical details
8	**Fig. 6 : qPCR-data**<br>***PR1***<br>t = 6.0766, df = 5, p-value = 0.0017<br>***PR2***<br>t = 8.5074, df = 5, p-value = 0.0003<br>***PR4***<br>t = 4.7361, df = 5, p-value = 0.0051<br>***PR5***<br>t = 6.0751, df = 5, p-value = 0.0017<br>***CAX3:***<br>t = 5.6604, df = 5, p-value = 0.0023<br>***PDF1.4***<br>t = 2.4657, df = 5, p-value = 0.0568<br>***VSP1***<br>t = 2.3015, df = 5, p-value = 0.0696<br>***MYC2***<br>t = 0.9019, df = 5, p-value = 0.4084
9	**Fig. 8: qPCR-data**<br>***PR1***

Non-flowering WT:

ANOVA1=aov(data$Expression~data$Treatment)

summary(ANOVA1)

	Df	Sum Sq	Mean Sq	F value	Pr(>F)
data$Treatment	2	347.8	173.89	66.75	2.55e-09
Residuals	19	49.5	2.61		

TukeyHSD(ANOVA1)

Tukey multiple comparisons of means

95% family-wise confidence level

Fit: aov(formula = data$Expression ~ data$Treatment)

$`data$Treatment`

	diff	lwr	upr	p adj
EF-C	9.8491	7.6574	12.0409	0.0000
F-C	3.6505	1.5283	5.7727	0.0009
F-EF	-6.1986	-8.3208	-4.0765	0.0000

Flowering WT:

ANOVA1=aov(data$Expression~data$Treatment)

summary(ANOVA1)

	Df	Sum Sq	Mean Sq	F value	Pr(>F)
data$Treatment	2	42.5	21.25	3.164	0.0629
Residuals	21	141	6.716		

TukeyHSD(ANOVA1)

Tukey multiple comparisons of means

95% family-wise confidence level

Fit: aov(formula = data$Expression ~ data$Treatment)

$`data$Treatment`

	diff	lwr	upr	p adj
EF-C	2.0268	-1.2393	5.2929	0.2827
F-C	-1.1974	-4.4635	2.0687	0.6315
F-EF	-3.2242	-6.4903	0.0419	0.0534

Flowering* svp-32*:

ANOVA1=aov(data$Expression~data$Treatment)

summary(ANOVA1)

	Df	Sum Sq	Mean Sq	F value	Pr(>F)
data$Treatment	2	42.12	21.061	6.537	0.00909
Residuals 21	15	48.33	3.222		

TukeyHSD(ANOVA1)

Tukey multiple comparisons of means

95% family-wise confidence level

Fit: aov(formula = data$Expression ~ data$Treatment)

$`data$Treatment`

	diff	lwr	upr	p adj
EF-C	3.4688	0.9508	5.9868	0.0073
F-C	0.9583	-1.8968	3.8135	0.6656
F-EF	-2.5105	-5.5200	0.4991	0.1099

Comparison of untreated controls:

Pairwise comparisons using t tests with non-pooled SD

data: data$Expression and data$Treatment

	SVP-C	WT-C
WT-C	0.00326	-
WT-FL-C	0.00585	0.00044

P value adjustment method: fdr

PR2

Non-flowering WT:

ANOVA1=aov(data$Expression~data$Treatment)

summary(ANOVA1)

	Df	Sum Sq	Mean Sq	F value	Pr(>F)
data$Treatment	2	163.86	81.93	30.76	1.1e-06
Residuals	19	50.61	2.66		

TukeyHSD(ANOVA1)

Tukey multiple comparisons of means

95% family-wise confidence level

Fit: aov(formula = data$Expression ~ data$Treatment)

$`data$Treatment`

	diff	lwr	upr	p adj
EF-C	6.636	4.420	8.852	0.000
F-C	4.701	2.555	6.847	0.000
F-EF	-1.934	-4.080	0.211	0.082

Flowering WT:

ANOVA1=aov(data$Expression~data$Treatment)

summary(ANOVA1)

	Df	Sum Sq	Mean Sq	F value	Pr(>F)
data$Treatment	2	10.92	5.459	1.507	0.244
Residuals	21	76.05	3.621		

TukeyHSD(ANOVA1)

Tukey multiple comparisons of means

95% family-wise confidence level

Fit: aov(formula = data$Expression ~ data$Treatment)

$`data$Treatment`

	diff	lwr	upr	p adj
EF-C	1.6518	-0.7465	4.0501	0.2156
F-C	0.8532	-1.5451	3.2515	0.6483
F-EF	-0.7986	-3.1969	1.5997	0.6833

Flowering* svp-32*:

ANOVA1=aov(data$Expression~data$Treatment)

summary(ANOVA1)

	Df	Sum Sq	Mean Sq	F value	Pr(>F)
data$Treatment	2	52.41	26.207	10.86	0.00105
Residuals 21	16	38.61	2.413		

TukeyHSD(ANOVA1)

Tukey multiple comparisons of means

95% family-wise confidence level

Fit: aov(formula = data$Expression ~ data$Treatment)

$`data$Treatment`

	diff	lwr	upr	p adj
EF-C	3.6857	1.5210	5.8503	0.0012
F-C	2.8385	0.5535	5.1235	0.0144
F-EF	-0.8472	-3.2742	1.5799	0.6476

Comparison of untreated controls:

pairwise.t.test(data$Expression, data$Treatment, p.adj="fdr", pool.sd=FALSE, var.eq=FALSE)

	SVP-C	WT-C
WT-C	0.3746	-
WT-FL-C	0.0029	0.0022

P value adjustment method: fdr

PR4

Non-flowering WT:

ANOVA1=aov(data$Expression~data$Treatment)

summary(ANOVA1)

	Df	Sum Sq	Mean Sq	F value	Pr(>F)
data$Treatment	2	70.92	35.46	8.35	0.00231
Residuals	20	84.93	4.25		

TukeyHSD(ANOVA1)

Tukey multiple comparisons of means

95% family-wise confidence level

Fit: aov(formula = data$Expression ~ data$Treatment)

$`data$Treatment`

	diff	lwr	upr	p adj
EF-C	3.7173	1.0191	6.4156	0.0063
F-C	3.6594	1.0526	6.2662	0.0054
F-EF	-0.0580	-2.7563	2.6403	0.9984

Flowering WT:

ANOVA1=aov(data$Expression~data$Treatment)

summary(ANOVA1)

	Df	Sum Sq	Mean Sq	F value	Pr(>F)
data$Treatment	2	0.18	0.0913	0.031	0.969
Residuals	21	60.97	2.9034		

TukeyHSD(ANOVA1)

Tukey multiple comparisons of means

95% family-wise confidence level

Fit: aov(formula = data$Expression ~ data$Treatment)

$`data$Treatment`

	diff	lwr	upr	p adj
EF-C	0.2135	-1.9339	2.3610	0.9660
F-C	0.0987	-2.0487	2.2462	0.9926
F-EF	-0.1148	-2.2622	2.0327	0.9901

Flowering* svp-32*:

ANOVA1=aov(data$Expression~data$Treatment)

summary(ANOVA1)

	Df	Sum Sq	Mean Sq	F value	Pr(>F)
data$Treatment	2	20.32	10.16	3.051	0.0795
Residuals 21	14	46.62	3.33		

TukeyHSD(ANOVA1)

Tukey multiple comparisons of means

95% family-wise confidence level

Fit: aov(formula = data$Expression ~ data$Treatment)

$`data$Treatment`

	diff	lwr	upr	p adj
EF-C	2.2184	-0.4387	4.8755	0.1087
F-C	2.2258	-0.7677	5.2193	0.1626
F-EF	0.0074	-3.0755	3.0903	1.0000

Comparison of untreated controls:

pairwise.t.test(data$Expression, data$Treatment, p.adj="fdr", pool.sd=FALSE, var.eq=TRUE)

data: data$Expression and data$Treatment

	SVP-C	WT-C
WT-C	0.56958	-
WT-FL-C	4.4e-05	0.00075

P value adjustment method: fdr

PR5

Non-flowering WT:

ANOVA1=aov(data$Expression~data$Treatment)

summary(ANOVA1)

	Df	Sum Sq	Mean Sq	F value	Pr(>F)
data$Treatment	2	25.57	12.786	6.112	0.00892

Residuals	19	39.75	2.092		

TukeyHSD(ANOVA1)

Tukey multiple comparisons of means

95% family-wise confidence level

Fit: aov(formula = data$Expression ~ data$Treatment)

$`data$Treatment`

	diff	lwr	upr	p adj
EF-C	2.6494	0.6650	4.6337	0.0082
F-C	0.5915	-1.2457	2.4287	0.6967
F-EF	-2.0579	-4.0422	-0.0735	0.0413

Flowering WT:

ANOVA1=aov(data$Expression~data$Treatment)

summary(ANOVA1)

	Df	Sum Sq	Mean Sq	F value	Pr(>F)
data$Treatment	2	7.41	3.706	1.048	0.371
Residuals	18	63.66	3.537		

TukeyHSD(ANOVA1)

Tukey multiple comparisons of means

95% family-wise confidence level

Fit: aov(formula = data$Expression ~ data$Treatment)

$`data$Treatment`

	diff	lwr	upr	p adj
EF-C	-0.4999	-2.9839	1.9841	0.8657
F-C	-1.4628	-4.0548	1.1292	0.3422
F-EF	-0.9629	-3.6331	1.7073	0.6348

Flowering* svp-32*:

ANOVA1=aov(data$Expression~data$Treatment)

summary(ANOVA1)

	Df	Sum Sq	Mean Sq	F value	Pr(>F)
data$Treatment	2	8.64	4.32	1.485	0.256
Residuals 21	16	46.55	2.91		

TukeyHSD(ANOVA1)

Tukey multiple comparisons of means

95% family-wise confidence level

Fit: aov(formula = data$Expression ~ data$Treatment)

$`data$Treatment`

	diff	lwr	upr	p adj
EF-C	1.4496	-0.9274	3.8266	0.2851
F-C	-0.0032	-2.5123	2.5060	1.0000
F-EF	-1.4527	-4.1179	1.2124	0.3611

Comparison of untreated controls:

pairwise.t.test(data$Expression, data$Treatment, p.adj="fdr", pool.sd=FALSE, var.eq=TRUE)

data: data$Expression and data$Treatment

	SVP-C	WT-C
WT-C	0.12430	-
WT-FL-C	0.00022	0.00142

P value adjustment method: fdr

CAX3

Non-flowering WT:

ANOVA1=aov(data$Expression~data$Treatment)

summary(ANOVA1)

	Df	Sum Sq	Mean Sq	F value	Pr(>F)
data$Treatment	2	198.08	99.05	109.6	09.3e-11
Residuals	18	16.27	0.90		

TukeyHSD(ANOVA1)

Tukey multiple comparisons of means

95% family-wise confidence level

Fit: aov(formula = data$Expression ~ data$Treatment)

$`data$Treatment`

	diff	lwr	upr	p adj
EF-C	7.1165	5.8197	8.4134	0.0000
F-C	5.6712	4.3743	6.9680	0.0000
F-EF	1.4454	2.7422	-0.1485	0.0278

Flowering WT:

ANOVA1=aov(data$Expression~data$Treatment)

summary(ANOVA1)

	Df	Sum Sq	Mean Sq	F value	Pr(>F)
data$Treatment	2	11.94	5.968	1.669	0.212
Residuals	21	75.08	3.575		

TukeyHSD(ANOVA1)

Tukey multiple comparisons of means

95% family-wise confidence level

Fit: aov(formula = data$Expression ~ data$Treatment)

$`data$Treatment`

	diff	lwr	upr	p adj
EF-C	1.7274	-0.6556	4.1104	0.1853
F-C	0.8585	-1.5245	3.2416	0.6413
F-EF	0.8689	-3.2529	1.5141	0.6346

Flowering* svp-32*:

ANOVA1=aov(data$Expression~data$Treatment)

summary(ANOVA1)

	Df	Sum Sq	Mean Sq	F value	Pr(>F)
data$Treatment	2	22.58	11.29	4.622	0.026
Residuals 21	16	39.08	2.443		

TukeyHSD(ANOVA1)

Tukey multiple comparisons of means

95% family-wise confidence level

Fit: aov(formula = data$Expression ~ data$Treatment)

$`data$Treatment`

	diff	lwr	upr	p adj
EF-C	2.16241	-0.01549	4.34031	0.05180
F-C	2.25990	-0.03909	4.55888	0.05439
F-EF	0.09749	-2.34443	2.53941	0.99417

Comparison of untreated controls:

```
pairwise.t.test(data$Expression, data$Treatment, p.adj="fdr", pool.sd=FALSE, var.eq=TRUE)
data:  data$Expression and data$Treatment
       SVP-C SVP-EF
SVP-EF 0.067 -
SVP-F  0.067 0.877
P value adjustment method: fdr
```

PDF1.4

Non-flowering WT:

ANOVA1=aov(data$Expression~data$Treatment)

summary(ANOVA1)

	Df	Sum Sq	Mean Sq	F value	Pr(>F)
data$Treatment	2	48.61	24.306	18.09	2.7e-05
Residuals	21	28.21	1.343		

TukeyHSD(ANOVA1)

Tukey multiple comparisons of means

95% family-wise confidence level

Fit: aov(formula = data$Expression ~ data$Treatment)

$`data$Treatment`

	diff	lwr	upr	p adj
EF-C	3.3987	1.9380	4.8595	0.0000
F-C	2.3713	0.9105	3.8320	0.0015
F-EF	-1.0274	-2.4882	0.4333	0.2029

Flowering WT:

ANOVA1=aov(data$Expression~data$Treatment)

summary(ANOVA1)

	Df	Sum Sq	Mean Sq	F value	Pr(>F)
data$Treatment	2	33.49	16.745	11.11	0.005
Residuals	21	31.65	1.507		

TukeyHSD(ANOVA1)

Tukey multiple comparisons of means

95% family-wise confidence level

Fit: aov(formula = data$Expression ~ data$Treatment)

$`data$Treatment`

	diff	lwr	upr	p adj
EF-C	2.6891	1.1418	4.2364	0.0007
F-C	2.2697	0.7224	3.8170	0.0037
F-EF	-0.4194	-1.9667	1.1279	0.7757

Flowering* svp-32*:

ANOVA1=aov(data$Expression~data$Treatment)

summary(ANOVA1)

	Df	Sum Sq	Mean Sq	F value	Pr(>F)
data$Treatment	2	9.186	4.593	7.489	0.00506
Residuals 21	16	9.813	0.613		

TukeyHSD(ANOVA1)

Tukey multiple comparisons of means

95% family-wise confidence level

Fit: aov(formula = data$Expression ~ data$Treatment)

$`data$Treatment`

	diff	lwr	upr	p adj
EF-C	1.4721	0.3808	2.5634	0.0082
F-C	1.3214	0.1694	2.4734	0.0237
F-EF	-0.1507	-1.3744	1.0729	0.9460

Comparison of untreated controls:

pairwise.t.test(data$Expression, data$Treatment, p.adj="fdr", pool.sd=FALSE, var.eq=TRUE)

data: data$Expression and data$Treatment

	SVP-C	WT-C
WT-C	0.0382	-
WT-FL-C	0.0817	0.0098

P value adjustment method: fdr

VSP1

Non-flowering WT:

ANOVA1=aov(data$Expression~data$Treatment)

summary(ANOVA1)

	Df	Sum Sq	Mean Sq	F value	Pr(>F)
data$Treatment	2	688.9	344.5	80.63	1.4e-10
Residuals	21	89.7	4.3		

TukeyHSD(ANOVA1)

Tukey multiple comparisons of means

95% family-wise confidence level

Fit: aov(formula = data$Expression ~ data$Treatment)

$`data$Treatment`

	diff	lwr	upr	p adj
EF-C	10.0489	7.4441	12.6538	0.0000
F-C	12.3346	9.7298	14.9394	0.0000
F-EF	2.2857	-0.3192	4.8905	0.0924

Flowering WT:

ANOVA1=aov(data$Expression~data$Treatment)

summary(ANOVA1)

	Df	Sum Sq	Mean Sq	F value	Pr(>F)
data$Treatment	2	643.0	321.5	56.65	3.46e-09
Residuals	21	119.2	5		

TukeyHSD(ANOVA1)

Tukey multiple comparisons of means

95% family-wise confidence level

Fit: aov(formula = data$Expression ~ data$Treatment)

$`data$Treatment`

	diff	lwr	upr	p adj
EF-C	9.9070	6.9045	12.9095	0.0000
F-C	11.8061	8.8036	14.8086	0.0000
F-EF	1.8991	-1.1034	4.9016	0.2700

***Flowering* svp-32:**

ANOVA1=aov(data$Expression~data$Treatment)

summary(ANOVA1)

		Df	Sum Sq	Mean Sq	F value	Pr(>F)
data$Treatment		2	3777.7	188.85	51.13	1.12e-07
Residuals	21	16	59.1	3.69		

TukeyHSD(ANOVA1)

Tukey multiple comparisons of means

95% family-wise confidence level

Fit: aov(formula = data$Expression ~ data$Treatment)

$`data$Treatment`

	diff	lwr	upr	p adj
EF-C	8.4901	5.8118	11.1683	0.0000
F-C	9.5915	6.7643	12.4186	0.0000
F-EF	1.1014	-1.9015	4.1043	0.6199

Comparison of untreated controls:

pairwise.t.test(data$Expression, data$Treatment, p.adj="fdr", pool.sd=FALSE, var.eq=TRUE)

data: data$Expression and data$Treatment

	SVP-C	WT-C
WT-C	0.074	-
WT-FL-C	0.074	0.718

P value adjustment method: fdr

MYC2

Non-flowering WT:

ANOVA1=aov(data$Expression~data$Treatment)

summary(ANOVA1)

	Df	Sum Sq	Mean Sq	F value	Pr(>F)
data$Treatment	2	45.07	22.537	18.12	2.68e-05
Residuals	21	26.12	1.244		

TukeyHSD(ANOVA1)

Tukey multiple comparisons of means

95% family-wise confidence level

Fit: aov(formula = data$Expression ~ data$Treatment)

$`data$Treatment`

	diff	lwr	upr	p adj
EF-C	2.3607	0.9551	3.7663	0.0010
F-C	3.2472	1.8416	4.6528	0.0000
F-EF	0.8865	-0.5191	2.2921	0.2719

Flowering WT:

ANOVA1=aov(data$Expression~data$Treatment)

summary(ANOVA1)

	Df	Sum Sq	Mean Sq	F value	Pr(>F)
data$Treatment	2	27.07	13.533	8.664	0.0018
Residuals	21	32.80	1.562		

TukeyHSD(ANOVA1)

Tukey multiple comparisons of means

95% family-wise confidence level

Fit: aov(formula = data$Expression ~ data$Treatment)

$`data$Treatment`

	diff	lwr	upr	p adj
EF-C	1.7945	0.2194	3.3696	0.0238
F-C	2.5281	0.9530	4.1032	0.0016
F-EF	0.7335	-0.8416	2.3087	0.4812

***Flowering* svp-32*:**

ANOVA1=aov(data$Expression~data$Treatment)

summary(ANOVA1)

	Df	Sum Sq	Mean Sq	F value	Pr(>F)
data$Treatment	2	21.23	10.613	4.538	0.0275
Residuals 21	16	37.42	2.339		

TukeyHSD(ANOVA1)

Tukey multiple comparisons of means

95% family-wise confidence level

Fit: aov(formula = data$Expression ~ data$Treatment)

$`data$Treatment`

	diff	lwr	upr	p adj
EF-C	1.9057	-0.2255	4.0370	0.0836
F-C	2.3600	0.1103	4.6097	0.0391
F-EF	0.4543	-1.9353	2.8438	0.8768

Comparison of untreated controls:

pairwise.t.test(data$Expression, data$Treatment, p.adj="fdr", pool.sd=FALSE, var.eq=TRUE)

data: data$Expression and data$Treatment

	SVP-C	WT-C
WT-C	0.13	-
WT-FL-C	0.67	0.13

P value adjustment method: fdr

Fig. 9A: Seed weight of non-flowering plants (seed production in climate chamber)

Simultaneous Tests for General Linear Hypotheses

Fit: lmer(formula = data$Seed_weight ~ data$Eggs * data$Feeding + (1 | Block), data = data)

Linear Hypotheses:

	Estimate	Std. Error	z value	Pr(>|z|)
(Intercept) == 0	85.13	12.14	7.010	2.38e-12
data$Eggsyes == 0	-23.57	17.17	1.373	0.168886
data$Feedingyes == 0	-62.55	17.17	3.642	0.000271
data$Eggsyes:data$Feedingyes == 0	53.55	24.72	2.166	0.030286

Signif. Codes: 0 ‘***’ 0.001 ‘**’ 0.01 ‘*’ 0.05 ‘.’ 0.1 ‘ ’ 1

(Univariate p values reported)

Fig. 9A: Seed weight of flowering plants (seed production in climate chamber)

Simultaneous Tests for General Linear Hypotheses

Fit: lmer(formula = data$Seed_weight ~ data$Eggs * data$Feeding + (1 | Block), data = data)

Linear Hypotheses:

	Estimate	Std. Error	z value	Pr(>|z|)
(Intercept) == 0	125.601	17.653	7.115	1.12E-12
data$Eggsyes == 0	-4.559	23.553	-0.194	0.847

	Estimate	Std. Error	z value	Pr(>|z|)
data$Feedingyes == 0	-5.994	25.119	-0.239	0.811
data$Eggsyes:data$Feedingyes == 0	10.494	37.069	0.283	0.777

Signif. Codes: 0 ‘***’ 0.001 ‘**’ 0.01 ‘*’ 0.05 ‘.’ 0.1 ‘ ’ 1

(Univariate p values reported)

Fig. S1: Seed weight of non-flowering plants (seed production in greenhouse)

Simultaneous Tests for General Linear Hypotheses

Fit: lmer(formula = data$Seed_weight ~ data$Eggs * data$Feeding + (1 | Block), data = data)

Linear Hypotheses:

	Estimate	Std. Error	z value	Pr(>|z|)
(Intercept) == 0	465.90	23.57	19.764	<2E-16
data$Eggsyes == 0	-71.99	32.85	-2.191	0.028420
data$Feedingyes == 0	-267.83	34.49	-7.764	8.22E.15
data$Eggsyes:data$Feedingyes == 0	180.73	48.80	3.704	0.000212

Signif. Codes: 0 ‘***’ 0.001 ‘**’ 0.01 ‘*’ 0.05 ‘.’ 0.1 ‘ ’ 1

(Univariate p values reported)

11 Table 2: C/N concentrations

Carbon (%):

ANOVA1= aov(data$Carbon~data$Treatment)

summary(ANOVA1)

	Df	Sum Sq	Mean Sq	F value	Pr(>F)
data$Treatment	3	8.27	2.758	0.554	0.65
Residuals	25	124.5	4.98		

TukeyHSD(ANOVA1)

Tukey multiple comparisons of means

95% family-wise confidence level

Fit: aov(formula = data$Carbon ~ data$Treatment)

$`data$Treatment`

	diff	lwr	upr	p adj
E-C	-0.7927	-4.2077	2.6223	0.9185
EF-C	-0.5847	-3.8998	2.7303	0.9616
F-C	-1.5148	-4.8299	1.8001	0.5977
EF-E	0.2079	-2.9689	3.3848	0.9978
F-E	-0.7221	-3.8990	2.4547	0.9229
F-EF	-0.9301	-3.9992	2.1390	0.8380

Nitrogen (%):

ANOVA1= aov(data$Nitrogen~data$Treatment)

summary(ANOVA1)

	Df	Sum Sq	Mean Sq	F value	Pr(>F)
data$Treatment	3	0.42	0.1401	0.732	0.542
Residuals	25	4.781	0.1913		

TukeyHSD(ANOVA1)

Tukey multiple comparisons of means

95% family-wise confidence level

Fit: aov(formula = data$Nitrogen ~ data$Treatment)

$`data$Treatment`

	diff	lwr	upr	p adj
E-C	0.2884	-0.3808	0.9576	0.6414
EF-C	0.0757	-0.5739	0.7253	0.9883
F-C	0.2689	-0.3807	0.9186	0.6696
EF-E	-0.2127	-0.8352	0.4098	0.7839
F-E	-0.0194	-0.6420	0.6031	0.9997
F-EF	0.1932	-0.4082	0.7947	0.8132

C:N ratio:

ANOVA1= aov(data$Ratio~data$Treatment)

summary(ANOVA1)

	Df	Sum Sq	Mean Sq	F value	Pr(>F)
data$Treatment	3	3.51	1.169	0.77	0.522
Residuals	25	37.95	1.518		

TukeyHSD(ANOVA1)

Tukey multiple comparisons of means

95% family-wise confidence level

Fit: aov(formula = data$Ratio ~ data$Treatment)

$`data$Treatment`

	diff	lwr	upr	p adj
E-C	-0.3294	-2.2148	1.5559	0.9626
EF-C	0.4983	-1.3318	2.3285	0.8762
F-C	-0.3130	-2.1432	1.5171	0.9648
EF-E	0.8277	-0.9261	2.5816	0.5725
F-E	0.0163	-1.7375	1.7702	0.9999
F-EF	-0.8114	-2.5058	0.8830	0.5609

12 Fig. 9B: Seed weight of non-flowering plants after recovery from larval feeding + cutting (seed production in greenhouse)

data: data$Seed.weight..mg by data$Treatment
t = -0.33133
df = 17
p-value = 0.7444
alternative hypothesis: true difference in means between group EF and group F is not equal to 0
95 percent confidence interval:
-53.97457 39.32279
sample estimates:
mean in group EF mean in group F
193.0111 200.3370

Supplementary Table S3 Weights of *Pieris brassicae* larvae on egg-laden (EF) or egg-free (F) non-flowering and flowering Arabidopsis. Additional data and statistical details. Larvae fed on leaves or flowers of previously egg-laden (EF) or egg-free (F) plants. Data were analysed either with a Student's *t*-test or with a Mann-Whitney U test, depending on the data distribution. Statistical test details (df; t or W) of each test are shown. In case of multiple pairwise comparison we *FDR*-adjusted the *P*-values. *P*-value in bold indicates significant differences between the two treatments (P < 0.05). The number of biological replicates (*N*, plants per treatment) is indicated. Table continues on next page. Exp.: Experiment, NFL: Non-flowering plants, FL: Flowering plants

Exp.	Plant stage (NFL/FL)	Plant tissue	Genotype	Light/dark cycle (hours)	Plant age (weeks)	Feeding period (days)	Treat-ment	Larval biomass (mg) means ± SE	df	t / W	*P* value	Statistical test	*N*
1	NFL	Leaf	Col-0	8/16	5	2*	F	0.5408 ± 0.0163	12	-5.3101	**0.0001**	*t*-test	7
							EF	0.3939 ± 0.0197					7
2	NFL	Leaf	Col-0	8/16	7	2*	F	0.3778 ± 0.0200	18	-3.0201	**0.0073**	*t*-test	10
							EF	0.2982 ± 0.0150					10
	NFL	Leaf	Col-0	8/16	7	6	F	5.0483 ± 0.2779	18	-2.6017	**0.018**	*t*-test	10
							EF	4.0520 ± 0.2340					10
3	FL	Leaf	Col-0	8/16	9	2*	F	0.3607 ± 0.0236	16	0.4419	0.6645	*t*-test	9
							EF	0.3781 ± 0.0284					9
	FL	Leaf	Col-0	8/16	9	5	F	1.1433 ± 0.0255	NA	22.5	0.119	U-test	9
							EF	1.1344 ± 0.0642					9
4	FL	Leaf	Col-0	8/16	12	2*	F	0.3842 ± 0.0200	14	-1.4376	0.1725	*t*-test	8
							EF	0.3394 ± 0.0211					8
	FL	Leaf	Col-0	8/16	12	14	F	156.4171 ± 6.2626	17	-0.5933	0.5607	*t*-test	9
							EF	149.6747 ± 8.4969					10
5	FL	Flower	Col-0	8/16	12	2*	F	0.5611 ± 0.0267	18	0.0513	0.9596	*t*-test	10
							EF	0.5631 ± 0.0264					10
6	FL	Leaf	Col-0	8/16 & 16/8	10	2*	F	0.3992 ± 0.0405	NA	30	0.2364	*U*-test	9
							EF	0.3604 ± 0.0223					10
	FL	Leaf	Col-0	8/16 & 16/8	10	12	F	95.6541 ± 6.4830	16	-0.4417	0.6646	*t*-test	9
							EF	90.3881 ± 9.1826					9
8	NFL	Leaf	Col-0	6/18	11	2**	F	0.4913 ± 0.0188	14	-3.8439	**0.0017**	*t*-test	8
							EF	0.3803 ± 0.0193					8
	FL	Leaf	Col-0	10/24	11	2**	F	0.2895 ± 0.0082	14	-0.0270	0.9788	*t*-test	8
							EF	0.2890 ± 0.0148					8
	NFL	Leaf	Col-0	8/16	7	2***	F	0.4591 ± 0.0330	14	-3.1594	**0.0069**	*t*-test	8
							EF	0.3306 ± 0.0187					8
	FL	Leaf	Col-0	16/8	7	2***	F	0.4060 ± 0.0218	14	-0.1676	0.8693	*t*-test	8

							EF	0.4000	±	0.0252					8
9	FL	Leaf	*svp-32*	8/16	7	2***	F	0.3163	±	0.0181	14	0.6963	0.4976	*t*-test	8
							EF	0.3162	±	0.0131					8
	NFL	Leaf	Col-0	8/16	7	6***	F	3.7248	±	0.4632	14	-2.9415	**0.0107**	*t*-test	8
							EF	2.0973	±	0.2306					8
	FL	Leaf	Col-0	16/8	7	6***	F	2.4061	±	0.3369	14	0.7467	0.4676	*t*-test	8
							EF	2.7523	±	0.2731					8
	FL	Leaf	*svp-32*	8/16	7	6***	F	1.4910	±	0.1211	14	0.6963	0.4976	*t*-test	8
							EF	1.6687	±	0.2057					8

* Data are presented in Fig. 1 ** Data are presented in Fig. 5 *** Data are presented in Fig. 7
